# Chromatography Coupled to High-Resolution Mass Spectrometry Analysis Reveals Flaws in van Krevelen Compound Class Assignments in DOM

**DOI:** 10.1021/acs.est.6c00809

**Published:** 2026-07-22

**Authors:** Maria G. Digernes, Alexander J. Craig, Tsz Yung Patrick Wong, Erik Bergström, Murat V. Ardelan, Jeffrey A. Hawkes

**Affiliations:** † Norwegian University of Science and Technology (NTNU) Department of Chemistry, Trondheim 7049, Norway; ‡ 8097 Uppsala University, Department of Chemistry for Life Sciences, BMC, Uppsala 75124, Sweden; § Swedish University of Agricultural Sciences (SLU), Department of Aquatic Sciences and Assessment, Uppsala 75007 Sweden

**Keywords:** natural organic matter, DOM, LC-HRMS, orbitrap, liquid chromatography–mass spectrometry, compound classes, van Krevelen, CRAM, lignin, peptide

## Abstract

High-resolution mass spectrometry (HRMS) is a powerful tool for determining molecular formulas in dissolved organic matter (DOM). In the biogeochemical field, it is common to assign DOM molecular formulas to prospective chemical classes based on elemental ratios alone, with these classes based on known biomolecules or empirical classifications (e.g., carboxyl-rich alicyclic molecules). However, accurate identification of molecular origin remains challenging due to DOM’s extreme isomeric diversity and the inability of HRMS to provide information about chemical connectivity, calling into question the accuracy of assignment or quantification. To address the lack of chemical connectivity gained from HRMS alone, we used liquid chromatography-HRMS to determine whether retention behavior is consistent with biochemical class assignment. Problematically, all examined classes displayed very broad retention profiles, indicating diversity of chemical functionality within their defined elemental ratios. Furthermore, retention behavior matched poorly with known biochemical class hydrophobicity data, particularly for “carbohydrates” and “peptides”. Additionally, differences in retention profiles of various compound classes and molecular formulas between terrestrial and marine samples show that molecular formulas are constituted by different groups of isomers between environments. We recommend an overhaul of standard practice in the use of HRMS data for biogeochemical investigation of DOM, relying less on assignment to prospective classes and more on chemical understanding, which can be developed through the implementation of chromatography and tandem MS methods using modern equipment and workflows.

## Graphical abstract

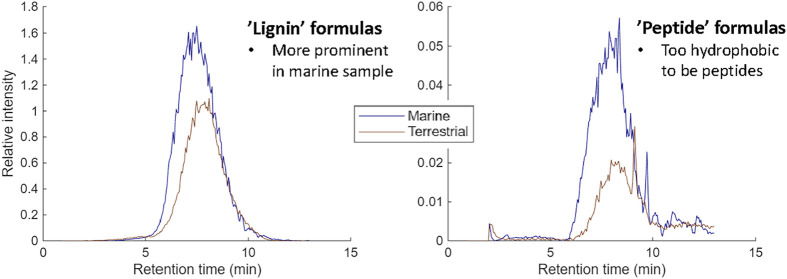

## Introduction

Dissolved organic matter (DOM) is a vast and complex mixture of organic compounds present in aquatic systems. In marine environments, it serves as a major active component of the global carbon cycle.^1^ One of the major methods for characterization of DOM is high-resolution mass spectrometry (HRMS), which has grown rapidly since the application of electrospray ionization in the early 2000s.^2^ Most research continues to be done using direct infusion introduction to ESI-HRMS, but the use of liquid chromatography (LC) in this field is growing and improving.^3–8^ The focus within direct infusion approaches has been on increased resolving power^9^ and data processing routines^10–13^ to achieve the best possible mass resolution and accurate formula assignment, frequently leading to tens of thousands of molecular formulas with associated relative intensities per sample. Following assignment of molecular formulas from the mass spectra, the assigned peaks are often plotted onto van Krevelen diagrams based on their H/C and O/C elemental ratios, aiding visualization of such complex data.^14,15^

There is a well-established tradition in DOM research of categorizing formulas into compound classes using defined elemental ratio boundaries, with these boundaries being adjusted over the last 25 years. Common classifications include biochemical classes such as carbohydrate-, tannin-, peptide-, and lipid-like compounds,^14,16–19^ and others include compound classes or features that have been empirically defined from DOM data such as carboxyl-rich alicyclic molecules (CRAM)^20^ and ‘island of stability’ molecular formulas.^21^ Notably, these compound class assignments are typically done based only on molecular formula data from direct infusion HRMS, and do not include information from chromatography (i.e., analyte polarity) or tandem MS (i.e., from collision-induced dissociation), which would typically be required for compound assignment in a field such as metabolomics.^22^ The problem with such simplified assignment criteria is the simple logical fallacy of the “converse error”. In the field, it is frequently assumed that because a compound class typically exists within a certain H/C and O/C range, all molecular formulas in that region can be ascribed to that compound class, and subsequently quantified and discussed in the context of environmental inputs or functions.

In some cases, research groups choose instead to use descriptive categories, such as “aliphatic compounds” for anything with H/C > 1.5,^23^ without designating the compounds into specific structural types such as “proteins” or “peptides”. This distinction is meaningful in establishing trends within the environment, as biomolecules have specific functions and sources, and incorrect assignment of HRMS data to such classes can lead to speculative and unfounded conclusions. Reinforcing these problems are response factor differences between functional groups and compound classes, which limits accurate quantification between molecular classes in ultracomplex mixtures such as DOM.^24^ Generally speaking, current DOM classification methods fail to resolve the key properties of organic matter that have climate relevance, particularly how factors such as molecular polarity and bioavailability control the contribution of DOM to oceanic CO_2_ sequestration and the efficiency of the biological and microbial carbon pumps.

LC coupled with HRMS (LC-HRMS) has proven to be a powerful method for studying DOM, offering advantages such as reducing competition among analytes at the ionization source and the incorporation of polarity characterization.^3,5,25–27^ Due to the ease of coupling chromatography online to Orbitrap mass analyzers, LC coupling has been standard practice for these spectrometers since near the beginning of their implementation in DOM research.^3,28^ In Fourier transform ion cyclotron resonance mass spectrometry (FT-ICR MS) analysis, ion trapping time is typically fixed, which complicates LC coupling due to changing ion populations during chromatographic separation. Nevertheless, LC-FTICR methods have been used in recent publications.^4,5,8,29–31^ Prior to the implementation of LC methods, work with FT-ICR-MS was restricted to time-consuming offline fractionation, which still led to important insights into mixture complexity in terms of isomeric diversity.^32–35^

In the present study, we employed LCMS to assess the retention behavior of various biomolecular compound classes that are commonly used in van Krevelen analysis within DOM research along a reversed-phase elution gradient. We also incorporated formulas associated with CRAM, riverine indicators (t-Peaks), and a stable subset of DOM compounds known as the island of stability (IOS).^21,36^ Additionally, we investigated small systematic regions of the van Krevelen space to show how effectively LCMS methods can differentiate isomer mixtures between DOM samples. Our aim was to evaluate the likelihood that certain biochemically inspired compound classes are assigned correctly and to probe what type of information can be gained using online chromatography that is otherwise lost in direct infusion.

## Materials and Methods

### DOM Samples

Marine samples from the northern Barents Sea region (76°N 31°E) were collected onboard research vessel *Kronprins Haakon* during a cruise in May 2021 at 30 m and at 316 m, which is approximately 10 m above bottom depth. Terrestrial samples were collected from three sites in Sweden: a humic lake named Plåten (59.8627°N, 18.5426°E), the end of the Munkedal river (58.4615 N, 11.6857 E), and a long water residence time lake (2–4 years) named Långsjö (59.4656°N, 17.3733°E). Suwannee River Fulvic Acid (SRFA) and Nordic Reservoir Natural Organic Matter (NRNOM) standards were obtained from the International Humic Substances Society (IHSS), batches 2S101F and 1R108N, respectively.

### Analysis of DOM Samples Using HPLC-HRMS

SRFA and the two marine samples were analyzed by reversed-phase high-performance liquid chromatography (HPLC), using an Agilent 1100 system with a C18 column (Phenomenex, Core–Shell C18 Kinetex 150 mm × 2.1 mm, 2.6 μm, 100 Å pore size). Mobile phase (A) consisted of LC-MS water with 0.1% formic acid, while mobile phase (B) was comprised of acetonitrile with 0.1% formic acid. Samples were diluted to 1 mg/mL in 5% acetonitrile solution and injected at 20 μL into the chromatographic system. A three-step gradient was used with a flow rate of 150 μL min^–1^. The initial mobile phase composition was 95% mobile phase A. At 1 min, mobile phase (B) was linearly increased to 95% at 10 min. At 10 min, mobile phase (B) was held isocratic at 95% for 2 min, before it was decreased to 5% until 15 min to equilibrate the column for the next injection.

Eluting material was analyzed in negative mode electrospray ionization (ESI) and an ion trap-orbitrap hybrid mass spectrometer (LTQ Velos Pro Orbitrap, Thermo Scientific). The ESI was set to a spray voltage of −3 kV; the inlet capillary was heated to 275 ^o^ C; the sheath gas flow rate was set to 28; and the S-lens level was at 68.7%. The mass resolution setting was set to 100,000, and automatic gain control was set to 1 × 10^6^ charges. Mass spectra were recorded between 150 and 1000 *m*/*z*. Internal calibration was performed in lock mass mode using a negative calibration solution with compounds capsaicin (304.1921 Da), fusidic acid sodium (515.3378 Da), and glycyrrhizic acid ammonium salt (821.3965 Da), which provided accuracy and precision of <2 ppm in the mass range of 120–822 Da.

### Analysis of Standard Compounds for Log *P* Calibration Using UPLC-HRMS

Reversed-phase ultraperformance liquid chromatography (UPLC) was applied with a Thermo Vanquish UHPLC system equipped with a C18 column (Phenomenex, Kinetex 1.7 μm C18, 150 mm × 2.1 mm, 100 Å pore size). Mobile phase (A) consisted of LC-MS grade water with 0,1% formic acid, while mobile phase (B) contained acetonitrile with 0.1% formic acid. Samples used for log *P* calibration were diluted to 1 μg/mL in 0.05% MeOH and injected at 10 μL into the chromatographic system. The same three-step gradient as the prior HPLC method was used with an adjusted flow rate of 500 μL min^–1^.

The compounds were ionized using negative mode ESI and a high-resolution mass spectrometer (Orbitrap Q Exactive, Thermo Scientific). ESI was set to a spray voltage of −3 kV; the inlet capillary was heated to 350 °C; the sheath gas flow rate was set to 35; the S-lens level was at 50; mass resolution was set to 140,000 at *m*/*z* 200. Mass spectra were recorded between 150 and 1000 *m*/*z*. This method was also used to reanalyze SRFA, and a further set of four terrestrial samples that are mentioned in the DOM samples section.

### Data Processing

Data processing was conducted using an in-house code written in Matlab (Version R2025a), which is available in the Supporting Information. Thermo .raw files were converted to mzXML using ReAdW, and signals <1000 counts, or with mass defects between 0.4 and 0.9, were removed as noise and interference. All other signals were first calibrated to mass 369.11911 at 7.5 min, and then all masses obtained in all spectra of all samples were stacked into one long mass list with corresponding intensities and sample/time identifiers (>28 million items). These masses were sorted into mass order and then clustered into center masses, allowing each sequential mass to join a center if within 4.5 ppm of the intensity-weighted center mass; otherwise, a new cluster center was initiated. After this cluster center routine, all signals with assigned clusters were placed into time bins of 3 s from 2 to 13 min (HPLC method, marine samples and SRFA), or 0.9 to 13 min (UPLC method, terrestrial samples and SRFA), creating intensity matrices for each sample with center masses as rows (34915 rows) and time bins as columns (242 columns). The center mass values were calibrated with a list of common DOM masses ranging from 154 to 673 Da (available in Supporting Information) using a fifth order polynomial function, and then formulas were assigned to the calibrated center masses between masses 120–700, H/C 0.3–2.2, O/C 0–1, N 0–3, S 0–1, ^13^C 0–1, DBE-O ≤ 10, valence neutral, when *N* > 0, (S + ^13^C) = 0, and S + ^13^C ≤ 1. An additional list of formulas from 2273 known metabolites, including cyanobacterial metabolites, was included in the formula list to increase coverage (including many peptides, etc.),^37,38^ with this list also available in the Supporting Information. The formula assignment routine led to 85% of the intensity from the average of SRFA and two marine samples being assigned. Masses without formulas assigned, ^13^C isotopologue peaks, and signals occurring in fewer than 14 spectra across the whole data set were excluded from further consideration, leading to a total of 10,172 formulas in the data set, and an intensity matrix of dimensions 10,172 × 242 for each sample.

Elemental ratio bounds for compound classes were averaged from 21 previous studies.^14,17,39–57^ Bounds for CRAM compounds were obtained from a molecular study on CRAM.^58^ Additionally, formulas for riverine input indicators were obtained from Medeiros et al.,^36^ and for island of stability compounds from Lechtenfeld et al.^21^

The octanol–water coefficient (*K*_ow_, or *P*) is defined as the partitioning of compound A in octanol ([A]_o_) and water ([A]_w_), and can be expressed as a logarithm (log* P*)­

$$\log_{10}P=\log_{10}\frac{{\left[A\right]}_{o}}{{\left[A\right]}_{w}}=\log_{10}{\left[A\right]}_{o}-\log_{10}{\left[A\right]}_{w}$$

In order to estimate log *P* of the data from the HPLC method described above, retention times from our most recent ultrahigh-performance liquid chromatography (UPLC) method were translated to retention times on the older HPLC method, as we no longer have that chromatography system or column. The retention time translation was done via retention times for all major peaks in SRFA (Figure SI1a). Retention times for a set of 11 commercially obtained compounds with experimental log *P* values were translated from the new method to the HPLC method, and a linear regression was established for retention time vs experimental log *P* (Figure SI1b)­

1 rt(min)=0.936⁡log⁡P+6.781

This relationship was used to determine approximate log *P* values for retention times of compound classes and molecular formulas. Note that this is an approximation: the training set is 11 compounds that do not necessarily share structural properties (and therefore retention characteristics) with DOM, and mapping retention times from one HPLC method to another comes with uncertainties.

## Results and Discussion

Our literature survey found many very highly cited research papers with slightly differing definitions of compound class boundaries (in H/C and O/C; Figure 1). As pointed out in other studies,^51,59^ actual known compounds from the named classes often fall outside of the regions drawn, which is already problematic. We decided to proceed with these average regions rather than the suggested regions that cover known compounds,^59^ as using a known-compound-driven approach would fall further into the converse error fallacy and would be less broadly applicable to the standard metrics used in the field. Furthermore, by using regions covering known compounds, many of the assigned formulas from DOM would not fall into any chemical class. The averaged regions we obtained overlap slightly in some cases but cover essentially the whole chemical space found in DOM. We also use definitions of CRAM,^20^ IOS,^21^ and t-peaks^36^ as empirically defined parts of DOM.

**Figure 1:**
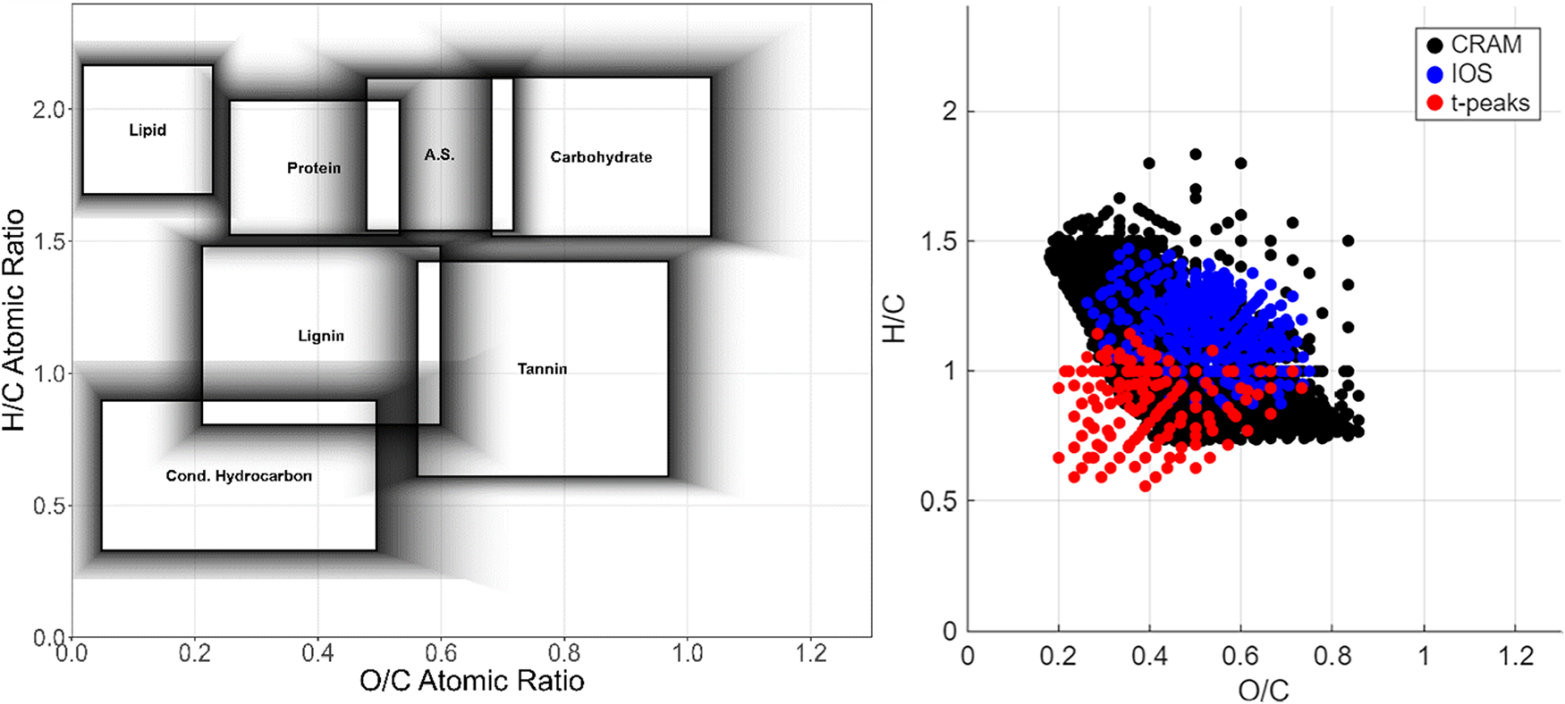
Compound class regions defined from the mean of 21 research articles (left), with standard deviations indicated with the gradient borders, and empirically defined classes (right). A.S. stands for amino sugars.

### Compound Class Information

The “lipid” region has been defined by low O/C ratios (0.01–0.35) and high H/C ratios (1.34–2.18), reflecting molecules with a high degree of saturation, large aliphatic functionalities, and occasional oxygen-containing functional groups. Although molecular formulas alone do not require these compounds to be carboxylic acids, their ionization in ESI-MS indicates this is likely, as at very high H/C ratios the incorporation of aromatic functionality (i.e., phenols) becomes difficult. At higher O/C ratios, but similar H/C ratios, is the “protein/peptide” region (herein, referred to only as “peptides”). Known peptides are composed of amino acids linked through amide bonds, with a mix of aliphatic and aromatic functional groups. Because the defined class does not necessarily include nitrogen,^51^ and the formula assignment routines often have a restricted number of nitrogen atoms, this compound class definition is rather loose and is often stated as “peptide-like”. At even higher O/C ratios are the “carbohydrate” region, defined by the highest O/C (0.56–1.23) and H/C ratios, and the “amino sugars” class, which is loosely defined by being slightly less oxygen-rich than carbohydrates. Amino sugars are rich in alcohols and may contain carboxylic acids, ketones, or aldehydes, and according to the biochemical compound class, must contain at least one nitrogen, similar to peptides (which must contain at least one per amino acid residue).

The less saturated compound classes (i.e., lower H/C ratios) are typically referred to as lignin (O/C < 0.60) and tannin (O/C > 0.56). Some studies also define an ‘unsaturated hydrocarbon’ region at very low oxygen numbers, but we exclude this class in this paper. The lower H/C ratios in these classes are typically ascribed to phenyl rings and carboxyl groups associated with vascular plant-derived compounds,^60^ but unsaturations can also come from the presence of aliphatic rings, where cyclized rings have fewer hydrogen atoms than their linear counterparts (i.e., hexane = C_6_H_14_, cyclohexane = C_6_H_12_). The monomers of lignin are hydroxy-propyl-phenyl compounds with aromatic methoxy substituents, where polymerized equivalents retain the aromatic functionality while incorporating a range of alcohols and cyclic aliphatic ethers. Biopolymeric tannin compounds are characterized by high O/C ratios (>0.5) and low H/C ratios (<1), where polymers include hydrolyzable tannins, which consist of sugar and gallic acid monomers, phlorotannins are oligomers of phloroglucinol, and condensed tannins are polymers of flavans. Condensed hydrocarbons are found at the lowest H/C values, and due to extremely high unsaturation, almost certainly contain extensive condensed aromatic rings, similar to black-carbon-like molecules.^61^

The empirically defined, DOM-related compound class of carboxyl-rich alicyclic molecules (CRAM) is hypothesized to constitute stable molecules that persist in marine environments for decades. CRAM spans the middle region of the van Krevelen diagram and has been proposed to derive from the oxidation of terpenoids^62^ or from oxidative dearomatization,^63^ but remains poorly defined.^27^ The CRAM class definition does in fact involve structural aspects,^20^ which have recently been revised,^63^ but most research only defines CRAM according to elemental ratios,^64^ leading to some investigations dealing with apparently labile portions of the CRAM class,^65^ which seems to be a contradiction.

Two further empirically defined groups of molecular formulas have been quite regularly used in the literature: the island of stability^21^ (IOS) and terrestrial peaks (t-peaks).^36^ Island of stability molecular formulas have been shown to be selectively enriched as DOM ages, and terrestrial peaks are enriched in river water and have been proposed as a proxy for determining terrestrial DOM input into ocean water.^66^ As a result, both are proposed as useful indicators for the extent to which DOM has been aged or diluted since it arrived in the ocean. Notably, both IOS and t-peaks (and all other compound classes) have been defined from direct infusion HRMS data, thus encompassing all isomers contributing to the molecular formula, and it generally has not been investigated whether stricter definitions (i.e., based on retention time or MS/MS characteristics) would provide more accurate proxies.

### Liquid Chromatography–Mass Spectrometry Data

Elution profiles for “peptide-like” peaks are visualized in Figure 2a by plotting the sum of the compound class formulas vs retention time. The retention profile data for all compound classes are summarized in Figure 2b, and corresponding profiles for all other classes are plotted in Figures SI2–SI12.

**Figure 2:**
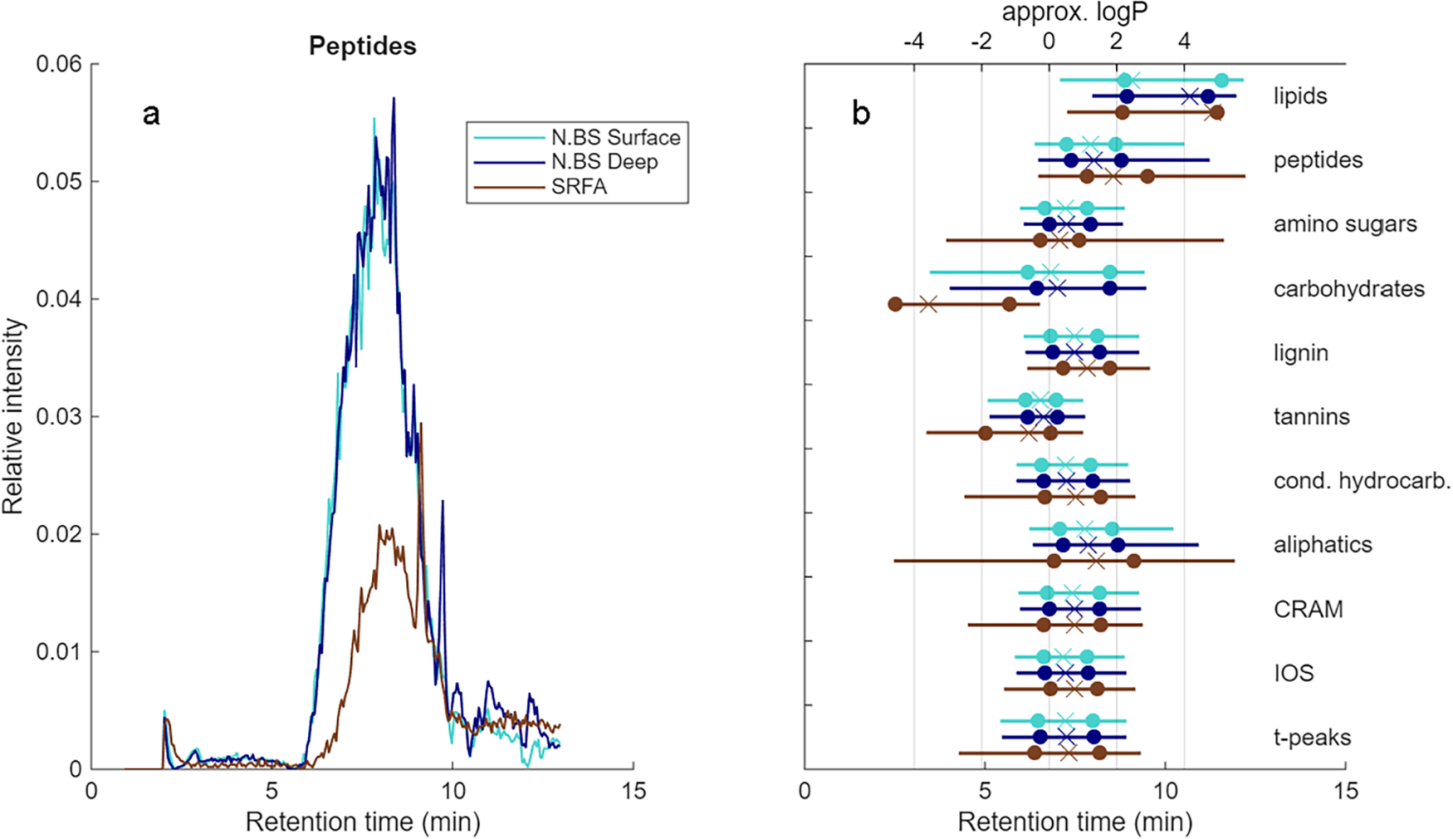
(a) Example retention profiles of a compound class (“peptides”) for the three samples. Relative intensity of the summed compound class is shown on the *y* axis, where a value of 100 signifies all summed intensity of the whole sample (summed across the whole retention profile). (b) Summary of retention profiles for all studied classes, where the 5th–95th percentiles of the data are shown as ranges, the 25th and 75th percentiles shown as points, and the median values of the retention profile shown as crosses.

The retention profiles of the compound classes generally followed an expected trend based on O/C ratio, where higher oxygen content leads to more polar compounds. This is clearly shown among the more saturated (high H/C ratio) compound classes, where "carbohydrates" were the first to elute, and lipids were the last. Generally speaking, the O/C ratio of a molecular formula is a good predictor for estimated log *P* (Figure SI13). More unsaturated compounds showed the same trend, with tannins eluting before lignin. There was a large overlap in retention between compound classes, which is not strictly problematic, although it is unlikely that significant portions of true peptides would be more lipophilic than any lipids. Indeed, the estimated log *P* range of  “peptides” in DOM here is rather surprising, where most of their intensity spans a log *P* of 0–5, and the majority exist above a log *P* of 1. Commercially available di- to penta-peptides^67^ typically span log *P* values of −3 to 2, with the vast majority having values below 1, and so if these formulas represent true peptides, then the peptide content of DOM is unusually hydrophobic in comparison to those found in purely biological systems. While this could be due to bias from SPE extraction, there is little ion intensity for peptide-like molecular formulas between 2 and 6 min (i.e., log *P* −3 to 0), while other formulas do elute here, indicating that this explanation is unlikely.

More troublingly, the assigned peptide molecular formulas detected between the SRFA and marine samples infrequently included nitrogen atoms (0, 1, 2, ≥3 N from 168, 29, 6, 10 total peptide molecular formulas, respectively). Considering that the vast majority of the assigned “peptides” were not nitrogen-containing, there is clearly a drastic problem in using this as an assignment category.^51^ Between this fundamental problem and the retention profile, it is difficult to say what is actually peptide-like about them. Due to the astonishing number of feasible molecular structures that can be drawn for molecular formulas near the middle of the van Krevelen diagram (numbering in the tens of millions per formula),^68^ it is much more likely that the majority of isomers constituting these molecular formulas are not related to peptides at all. Notably, the assigned “peptides” made up a higher proportion of the extracted DOM in marine samples compared to SRFA (Table 1), and on average were more hydrophilic, indicating fundamental chemical differences between environments. The problem with the lack of nitrogen-containing formulas was shared by the “amino sugar class”: 124 of the 160 formulas detected in this class between the SRFA and marine samples did not contain any nitrogen, excluding them as true amino sugar candidates.

**Table 1:** Percentage Contributions of Different Compound Classes to All LCMS Intensity (i.e., Summed Intensity across the Retention Profile)^a^

	N.BS surface %	N.BS deep %	SRFA %
lipids	0.0	0.0	0.0
peptides	2.4	2.6	1.2
amino sugars	0.8	0.7	0.3
carbohydrates	0.1	0.1	0.3
lignin	75.4	75.3	49.7
tannins	24.9	24.4	41.8
cond. hydrocarb.	0.7	0.7	3.4
Aliphatics (H/C > 1.5)	4.2	4.3	2.0
CRAM	67.1	67.0	60.7
IOS	45.3	44.6	24.8
t-peak	2.1	2.1	7.8

a Note that the regions overlap (Figure 1) and do not cover the whole chemical space, so these percentages can sum to more or less than 100%.

There are far more constraints on molecular structure as the O/C ratio tends toward 0 and 1, limiting the feasible isomer count and potential for structural diversity for lipids and carbohydrates.^69^ However, the range in retention times remains quite large, suggesting a significant diversity in backbone structure and chemical functionality. The retention time range also varied between marine and terrestrial DOM types (Figure 2b). Specifically, carbohydrates in SRFA began eluting somewhat earlier, with 25% of intensity elution reached by 2.5 min, but for the surface and deep marine samples, 25% of intensity elution was reached at 6.2 and 6.4 min, respectively, by which point nearly all of the carbohydrates in SRFA had eluted. Note that intensity does not necessarily correspond to concentration in this method. This pattern indicates greater polarity heterogeneity among carbohydrate-designated compounds of terrestrial origin. The elution of most carbohydrate intensity after 5 min for marine samples in this reversed-phase chromatography method indicates that they likely represent compounds with higher hydrophobicity than common sugars (e.g., glucose log *P* ∼ −1.7, glucuronic acid ∼−3.13). For instance, lipopolysaccharides or glycoproteins derived from components of cell membranes^62^ would show early elution on a hydrophobic stationary phase, but compounds such as these could not account for the highest O/C ratio formulas assigned in DOM. The poor ionization efficiency of true carbohydrates under electrospray ionization (ESI)^20,58^ and low extraction efficiency on PPL-SPE sorbent^70^ would also contribute to their relatively low proportion (<0.5%) in both marine and SRFA samples (Table 1), despite being prominent when studying seawater DOM with NMR.^71,72^

Moving to the more unsaturated compound classes (lignin, tannin and condensed hydrocarbons), we found that lignin and tannin dominated all three samples in terms of the ion intensity count (Table 1). While for SRFA the two classes were close to evenly split, lignin content in marine samples was three times higher than tannin content. Neither compound class would be traditionally expected in marine environments, being derived from terrestrial plant materials, but it should be noted that both categories are typically poorly explained in the DOM literature.^20,58^ SRFA showed higher polar variability within the tannin compound class compared to the marine samples (Figure 2b), and notably displayed a feature between 2.5 and 5.2 min, which was absent in marine samples, and may be useful in tracing terrestrial input.^73^ Critically, naturally occurring tannin and lignin polymers and the majority of their monomers are highly aromatic, but NMR studies of marine DOM have routinely shown a low prevalence of aromatic functionality. The apparent dominance of lignin within marine samples in the context of known structures in these classes is as such impossible, further reducing the utility of these compound class definitions.

It is important to point out that our analysis focuses on false positive assignments: those for which a compound class like “peptide” is assigned without sufficient evidence. It is also important to consider and think about false negatives in the same analytical pipeline, i.e., peptides that are truly in the sample, but are not analyzed and contributing to the data, and this may be due to inefficient extraction (e.g., with SPE), inefficient retention on column or ionization in ESI, poor ion stability resulting in fragmentation, or incomplete resolution or formula assignment within the correct atomic constraints (e.g., not enough nitrogen or sulfur). This type of analysis pipeline (SPE-LC-ESI-HRMS) cannot rule out the presence of peptides, carbohydrates, lignin, etc. in the aquatic environment; clearly, these compound classes are present, as evidenced by other analytical techniques. The problem is that the analytical window used in this method is inappropriate for their analysis, and there is a high risk of false positives using only DI-HRMS analysis with the traditional use of van Krevelen-based compound class assignment.

### Molecular Formula-Based Biogeochemical Indices

In our LC-HRMS experiments, we found that the t-peaks had a very stable background concentration in the marine samples (i.e., were essentially identical at the two marine depths), and they were proportionally higher in intensity than the condensed hydrocarbon class in the marine samples compared to SRFA (Figure 3). Notably, both tannin and condensed hydrocarbon classes were particularly absent in marine samples at the most hydrophilic end (i.e., before 3 min), indicating that a combined retention time and formula-based criteria may serve best for determination of the content of removable terrestrial DOM.^73^ It has been noted recently that large amounts of terrestrial DOM may continue to be present in marine systems, contributing to long-term DOM storage in the oceans, either through direct preservation due to recalcitrance,^73,74^ or because of modification (i.e., through bleaching), which can hide or camouflage terrestrial DOM within the marine pool.^75^ The tracing of such recalcitrant terrestrial DOM is likely to be improved by including chromatographic and MS/MS information.^26,73^ Improving methods for tracing tDOM in the ocean is essential for determining whether coastal systems function as climate buffers or amplifiers. Understanding how the polarity and other characteristics of tDOM affect its bioavailability and recalcitrance has significant implications for ecosystem carbon fluxes and long-term carbon sequestration. These factors serve as foundational feedbacks for climate processes, biodiversity dynamics, and the blue carbon budget and strongly influence global carbon models.

**Figure 3:**
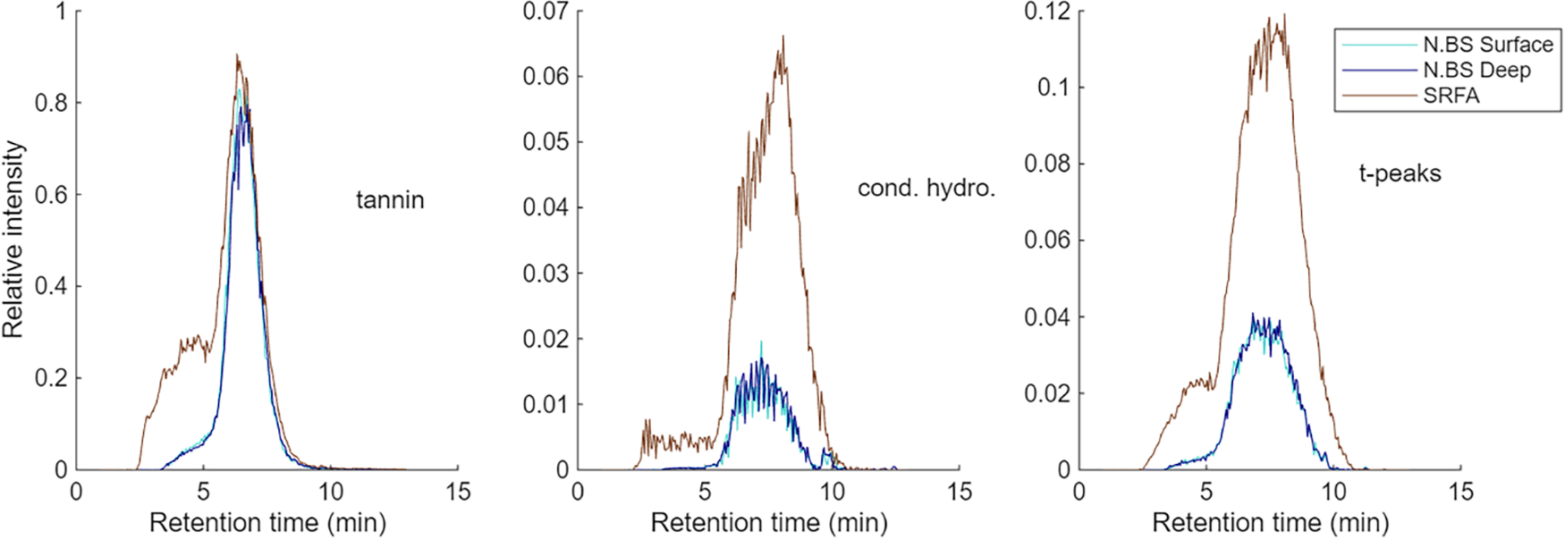
Unsaturated compound classes and t-peaks, potentially of use for tracing terrestrial DOM. Relative intensity of the summed compound class is shown on the *y* axis, where a value of 100 signifies all summed intensity of the whole sample (summed across the whole retention profile). Cond. hydro. refers to condensed hydrocarbons.

A total of 135, 134, and 139 t-Peaks were detected in the surface, deep N.BS, and SRFA samples, compared with 184 peaks in the full list. SRFA was composed of a higher proportion (7.8%) of t-Peaks in terms of total intensity than the marine samples (2.1% N. BS surface and N.BS deep) (Table 1), which confirms the selective removal or dilution of these compounds in the marine environment. Removal of t-peaks may occur through biodegradation, as shown in incubation experiments in a sub-Arctic fjord,^76^ or through photochemical oxidation to CO_2_, which has been observed for more aromatic compounds.^77^

Concerningly, “CRAM” compounds were relatively more abundant in the terrestrial sample (despite being largely considered as marine-derived),^64,78^ while “lignin” was relatively more abundant in marine environments, despite being defined from terrestrial plants (Figure 4). This likely indicates that the compounds found in aquatic environments are much more chemically and source diverse than these names suggest. We recommend that the term “lignin” or “lignin-like” should not be used further in this type of DOM characterization, as the potential for diversity in molecular structure at these elemental ratios is far too high to be constrainable to a single type of structure, and the evidence suggests that a large amount of DOM with these formulas in marine samples are not sourced from degraded plant structure material on land. CRAM appears to be a useful research direction for investigating RDOM formation, but our efforts to date suggest that the simplest type of proposed CRAM structure (alicyclic cores with only external carboxylate, ketone, and alcohol groups) is not an important contributor to terrestrial or marine DOM.^26,79^ There are two major problems associated with the use of the “CRAM” terminology currently, with the first being that, like “peptides”, it is a logical fallacy to state that if an assigned formula sits in the same region that CRAM would, the compound must be a CRAM.^64,65^ The second is that CRAM is currently too poorly defined. While likely structures have recently been updated,^26,63^ there are significant limitations in the understanding of structures that actually comprise the carboxyl-rich component of DOM. Assignments of ‘CRAM’ certainly require more than just formula assignment, and at minimum require the examination of MS2 fragmentation patterns or retention time information.

**Figure 4:**
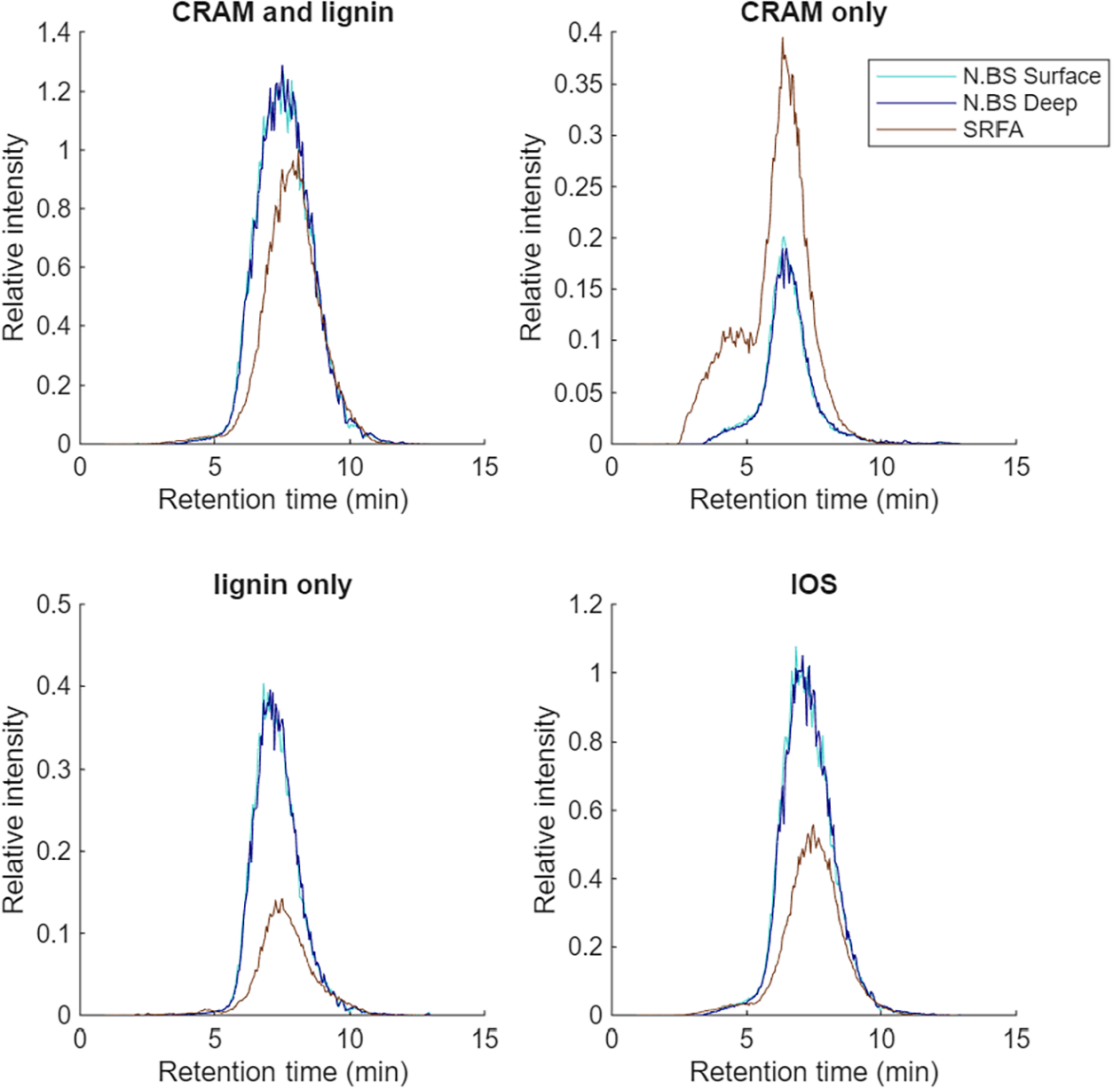
Retention profiles for compounds defined as both CRAM and lignin (e.g., overlapping in both definitions), only as CRAM (not lignin), only as lignin (not CRAM), and IOS, in the three samples analyzed. Relative intensity of the summed compound class is shown on the *y* axis, where a value of 100 signifies all summed intensity of the whole sample (summed across the whole retention profile).

The island of stability (IOS) index refers to molecular formulas empirically determined to be of high stability in the marine environment, but with unknown structures.^21^ In SRFA, IOS compounds showed a slightly wider range in polarity with some eluting as early as 4 min (Figures 2b and 4). Additionally, there was a disproportionately higher presence of IOS compounds in marine samples (45% N.BS surface and N.BS deep) compared to SRFA (25%) (Table 1). This raises the possibility of their selective enrichment as DOM is modified along the land-to-ocean aquatic continuum, instead of there being a purely marine source of these compounds. However, as with all of these classes and indeed molecular formulas, the chemical complexity of DOM is vast, and the isomers present in IOS formulas may differ between marine and terrestrial environments. Notably, there is a tendency for all stable compound classes to have more averaged retention times (i.e., not extremely early or late) in marine samples compared to terrestrial DOM elution profiles, which might reflect long-term molecular selection processes favoring intermediate polarity structures in the marine environment.

### Implications and Possible Alternatives

Here, we have shown that the use of defined regions in van Krevelen space based on prospective compound classes is fundamentally flawed. Basic chemical logic can immediately rule out the majority of DOM’s “peptide” molecular formulas as peptide-like based solely on the absence of nitrogen atoms, a problem which is shared with DOM’s so-called “amino sugar” region. LC-MS methods help to reinforce this by highlighting the mismatch in polarity characteristics between known di- to penta-peptides and the “peptide” formulas in DOM, a problem that carries over to the ‘carbohydrate’ region. While differences in retention profiles exist between compound classes for marine and terrestrial samples, there is a clear overlap of the hydrophobic regions (i.e., rt = 5 to 10 min) of ‘tannin-like’ and ‘lignin-like’ compounds between environments, with these two regions accounting for over 90% of the total signal intensity in all three examined samples. This, again, is problematic when taken alongside the deficiency of aromatic functionality in marine DOM, which is known from NMR spectroscopy. If it is the case that these hydrophobic isomers have different functional group compositions but coincidentally similar retention profiles between environments, it should be immediately clear that this type of MS-based compound class identification is not a useful tool in understanding biogeochemical inputs. Furthermore, the major difference for the abundant “lignin” and “tannin” groups of formulas is an early eluting peak within tannin for SRFA (rt = 2.5 to 5.2 min), which perhaps could be speculated to contain the “aromatic” functionality of this group of formulas. However, this peak is also clearly observed in the retention profile of the empirical “CRAM-like” class (also in SRFA), and reinforces the inability for MS-based analysis to meaningfully distinguish between biogeochemical inputs and their chemical structures.

In this study, two marine samples from the same station but different depths were found to be highly similar to each other, across the LC separation dimension as well as based only on the intensity of mass spectral peaks. These were compared to a single terrestrial sample, the reference standard SRFA. In order to examine the applicability of this reference material as a good representation of terrestrial DOM, we additionally compared it to four other terrestrial samples, three of which were PPL extracts like the marine DOM (Figures SI14–S15). This investigation showed that SRFA is broadly very similar to other terrestrial samples for every compound class and empirical molecular formula-based index, with “peptides” as the main exception, as the "peptides" were somewhat lower in abundance and more hydrophobic in SRFA than the other samples. Generally, the terrestrial sample with a high water residence time had chromatographic retention profiles more similar to the marine sample, likely due to being more biogeochemically processed.^23^ Note that we promote the use of reference mixtures in DOM analytical papers, especially those based on method development, as they facilitate comparison between laboratories.^80–82^

As an alternative to the use of compound classes, we propose that smaller van Krevelen regions should be compared empirically and systematically. In Figure 5, we demonstrate this idea by gridding van Krevelen space into increments of 0.05 for O/C and 0.1 for H/C. Extracted ion chromatograms are shown for the sum of assigned formulas in four adjacent cells (at H/C 1.1–1.2 and increasing O/C). In all four cases, both marine samples showed almost identical retention profiles and ion intensities. However, for SRFA, as the O/C ratio increased, isomers became lower in intensity and more hydrophobic relative to the marine samples, even while molecular formula composition remained similar. While SRFA was more hydrophobic on average than the marine samples within these molecular formula regions, it was more hydrophilic across the full sample. This was almost entirely due to the prevalence of the hydrophilic (rt = 2.5–5.2 min) ‘tannin-like’ peak (Figures 2b and 3). It is of course possible to investigate the retention profiles of individual molecular formulas, and future research will aim to cluster these into families of compounds that show similar environmental trends in terms of their retention profiles alongside their changing abundance between samples.^83^

**Figure 5:**
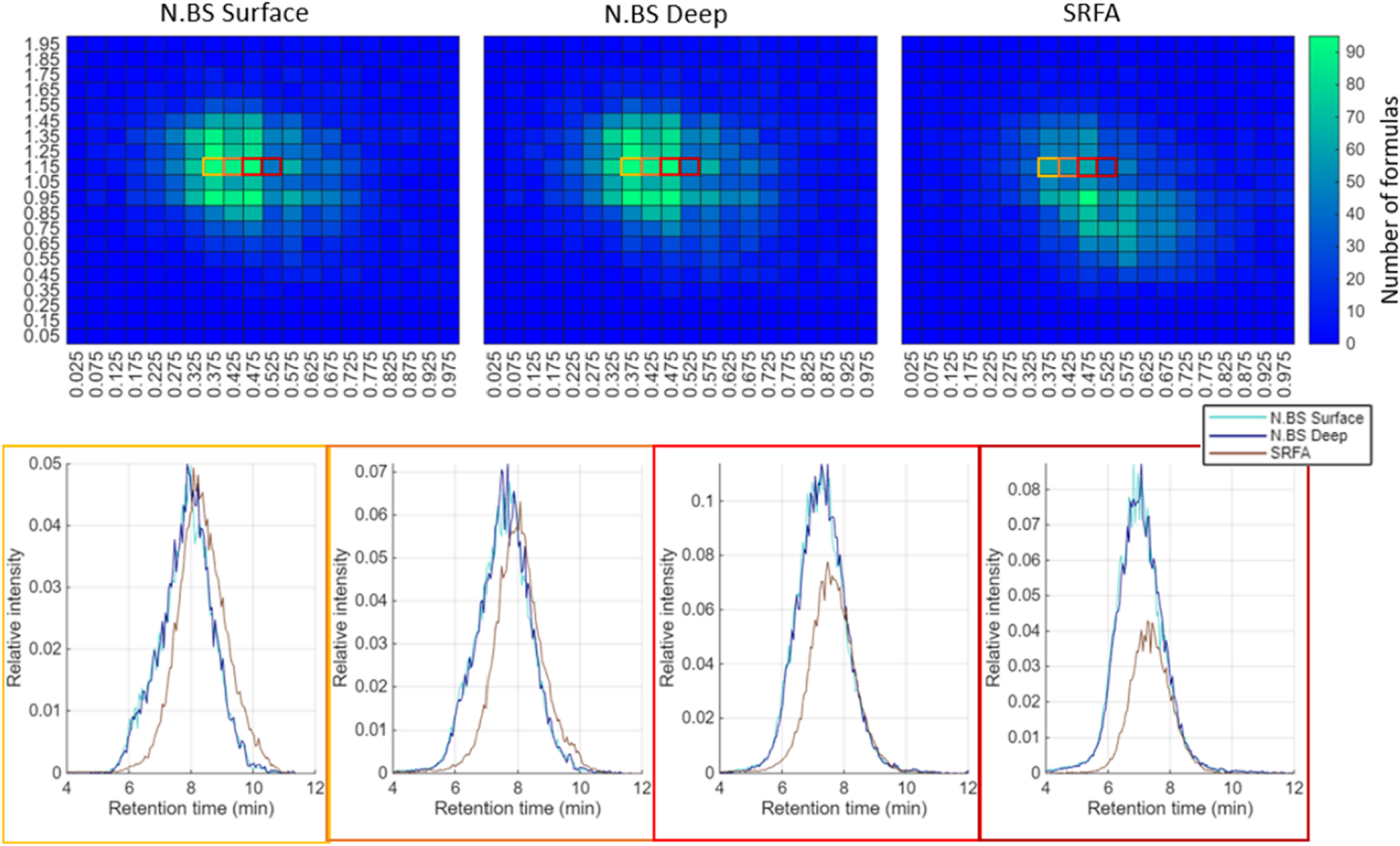
Top: Gridded van Krevelen diagrams for the three samples showing the number of formulas in each cell (0.05 O/C and 0.1 H/C) as a heat map. Bottom: four cells are selected for plotting the sum of all formulas as extracted ion current chromatograms, with border colors corresponding to colored squares in the van Krevelen diagrams.

Ultimately, HRMS, and in particular LC-HRMS, remains a powerful tool for the investigation of compositional differences between different DOM samples. However, in the context of biogeochemical compound classes, these approaches can only show correlation and not causation, and those seeking to quantify compound class regions, even those based on empirical metrics (i.e., “CRAM-like”), fail to understand the limitations of mass spectrometry. Empirically defined collections of peaks, such as IOS and t-peaks, may be useful in tracing terrestrial DOM or for providing metrics about DOM aging, but they provide no insight into the chemical structure or the ecological source of material, which are often key aims of HRMS studies. Additionally, these peaks have previously only been defined from direct infusion data, and the chromatographic distribution of their isomers has not been previously disclosed or considered as part of their ecological significance. Accurate chemical understanding is required for the delineation of the environmental processes surrounding DOM, as well as to determine how anthropogenic inputs affect its composition and behavior.

We believe that it is imperative that van Krevelen analysis is decoupled from implied biogeochemical compound classes if robust chemical understanding is to be achieved. Furthermore, we urge the wider adoption of online LC methods for the HRMS analysis of DOM, a development that has already seen great progress in recent years,^5,7,8,73^ and for biogeochemical interpretation to be based on systematic and empirical molecular formula regions.

## Supplementary Material



## Data Availability

The Matlab code and raw mzXML files used are available at a Zenodo repository: https://zenodo.org/records/20827286.

## Notes

Details and figures regarding translation of retention times between two chromatographic methods, figures showing the chromatographic distributions of all compound classes mentioned, weighted average retention time of each measured formula shown on van Krevelen space for a marine and terrestrial sample, and further data for a range of terrestrial samples (PDF)
