# Supplementary material for: Chromatography Coupled to High-Resolution Mass Spectrometry Analysis Reveals Flaws in van Krevelen Compound Class Assignments in DOM
Source: Environ Sci Technol. 2026 Jul 22;60(30):20951–62. doi: 10.1021/acs.est.6c00809 (PMC13449886; doi:10.1021/acs.est.6c00809)
Supplement: Supplementary file 1 [file es6c00809_si_001.pdf]

Supplementary Information for:

Chromatography coupled to high resolution mass spectrometry analysis reveals flaws in van Krevelen compound class assignments in DOM

Maria G. Digernes[1], Alexander J. Craig[2], Tsz Yung (Patrick) Wong[2,3], Erik Bergström[2], Murat V. Ardelan[1], Jeffrey A. Hawkes[2*]

[1] Norwegian University of Science and Technology (NTNU), Department of Chemistry, Trondheim 7049, Norway

[2] Uppsala University, Department of Chemistry for Life Sciences, BMC, Uppsala 75124, Sweden

[3] Swedish University of Agricultural Sciences (SLU), Department of Aquatic Sciences and Assessment, 750 07, Uppsala, Sweden

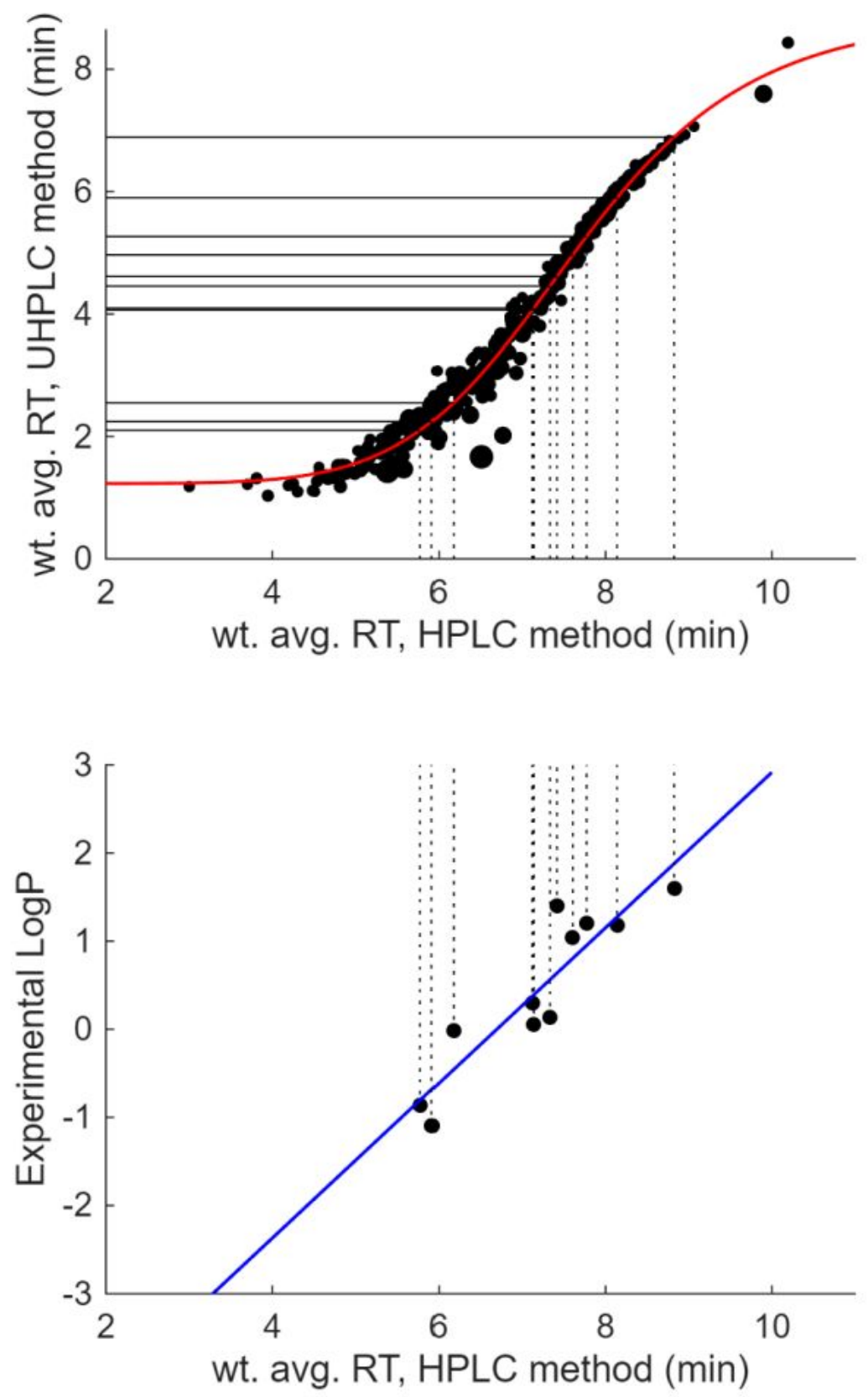

Figure SI1: Top: Translation of retention times from new method (y axis) to the HPLC method from the present study (x axis). Shown as solid dots are weighted average retention times of the most intense peaks from SRFA (those with summed intensity >20% of the maximum summed intensity), and the red regression is a 4-parameter logistic (Hill) function of the type:

$$y = d + \frac{a-d}{1+\left(\frac{x}{c}\right)^b}$$

Horizontal lines correspond to the retention times of 11 commercially available compounds with experimental logP values, and their translated (predicted) retention times on the HPLC method are shown as dotted vertical lines, which are also shown in the bottom panel. Bottom: Predicted retention times of the 11 compounds plotted against their experimental logP values, leading to a linear regression shown as a blue line.

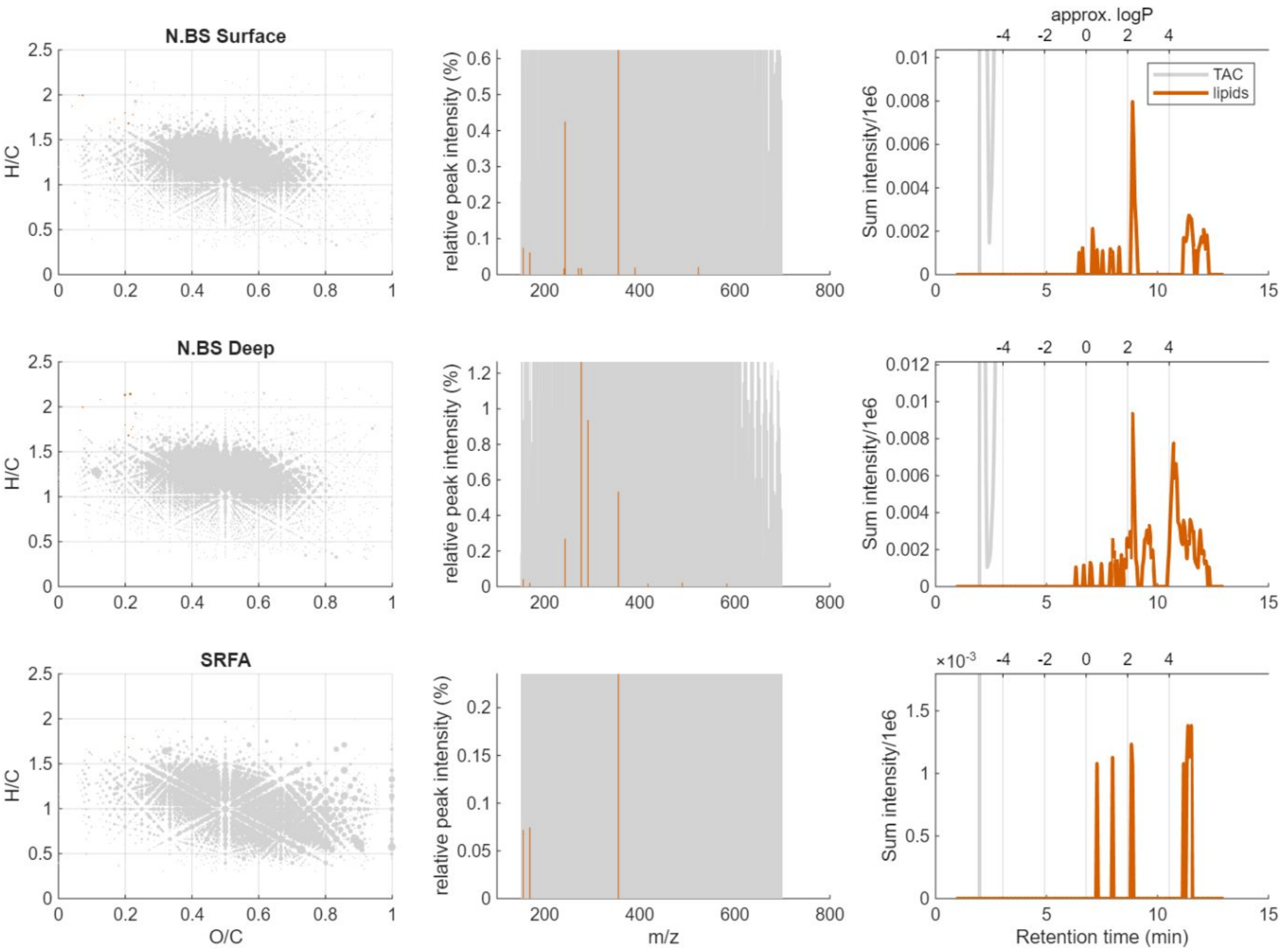

Figure SI2: Van Krevelen diagrams, mass spectra and retention profiles for the 'lipid' compound class, superimposed onto all assigned formulas, assigned mass spectrum, and total ion current, respectively. The data are shown for all three samples, and in some cases the mass spectrum or TIC is out of scale, but can be seen in other figures (SI2-12).

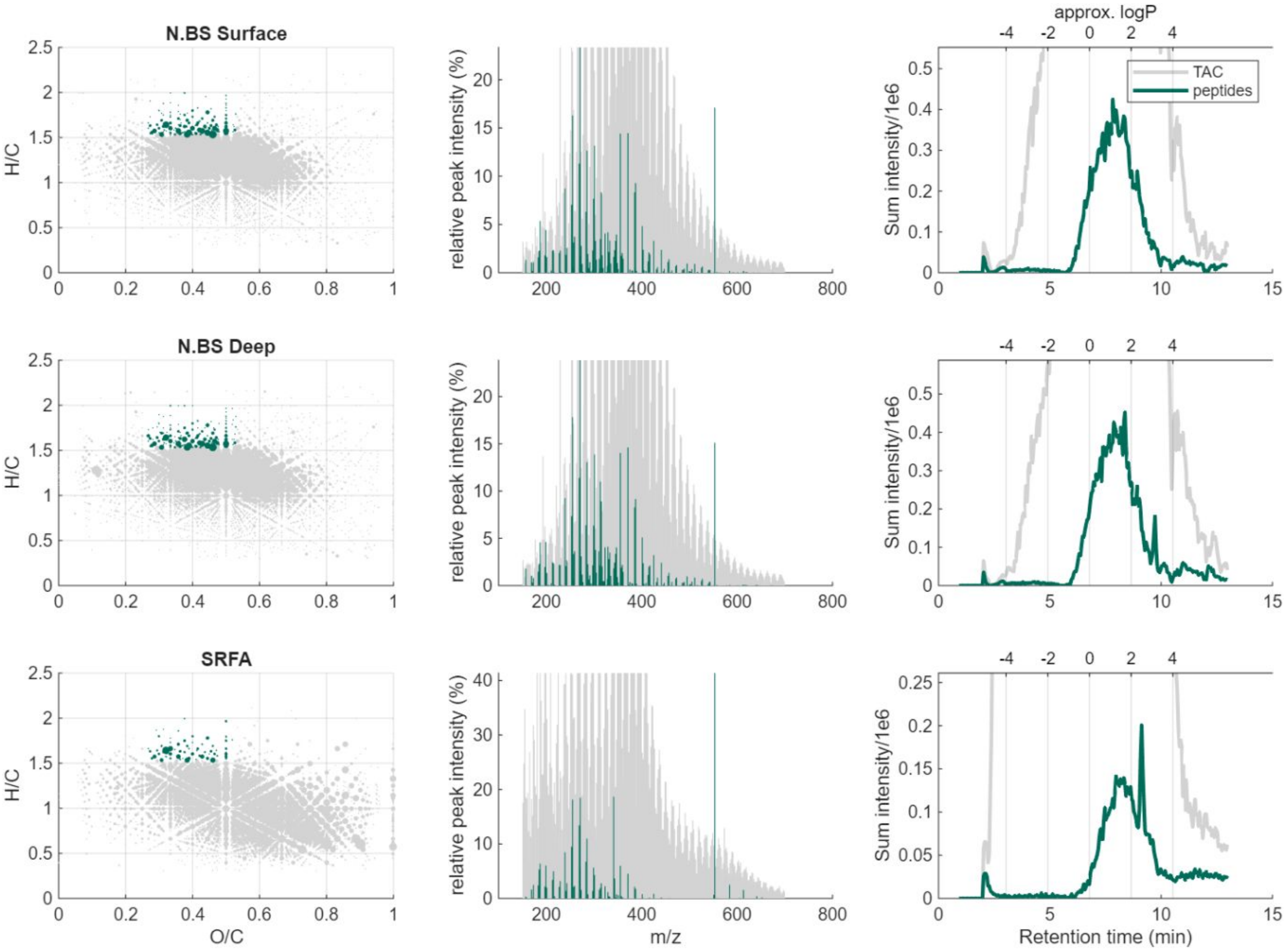

Figure SI3: Van Krevelen diagrams, mass spectra and retention profiles for the 'peptide' compound class, superimposed onto all assigned formulas, assigned mass spectrum, and total ion current, respectively. The data are shown for all three samples, and in some cases the mass spectrum or TIC is out of scale, but can be seen in other figures (SI2-12).

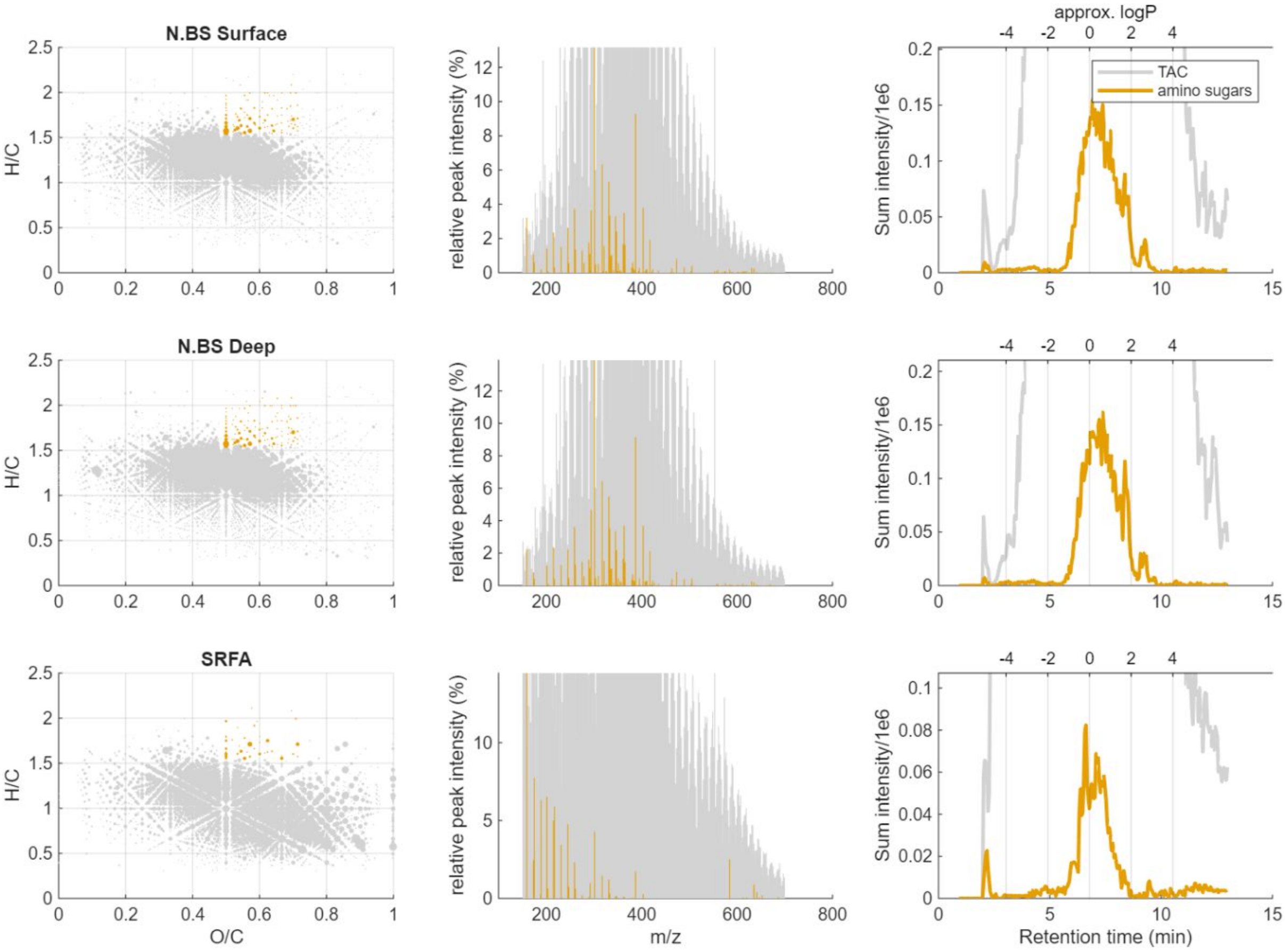

Figure SI4: Van Krevelen diagrams, mass spectra and retention profiles for the ‘amino suugar’ compound class, superimposed onto all assigned formulas, assigned mass spectrum, and total ion current, respectively. The data are shown for all three samples, and in some cases the mass spectrum or TIC is out of scale, but can be seen in other figures (SI2-12).

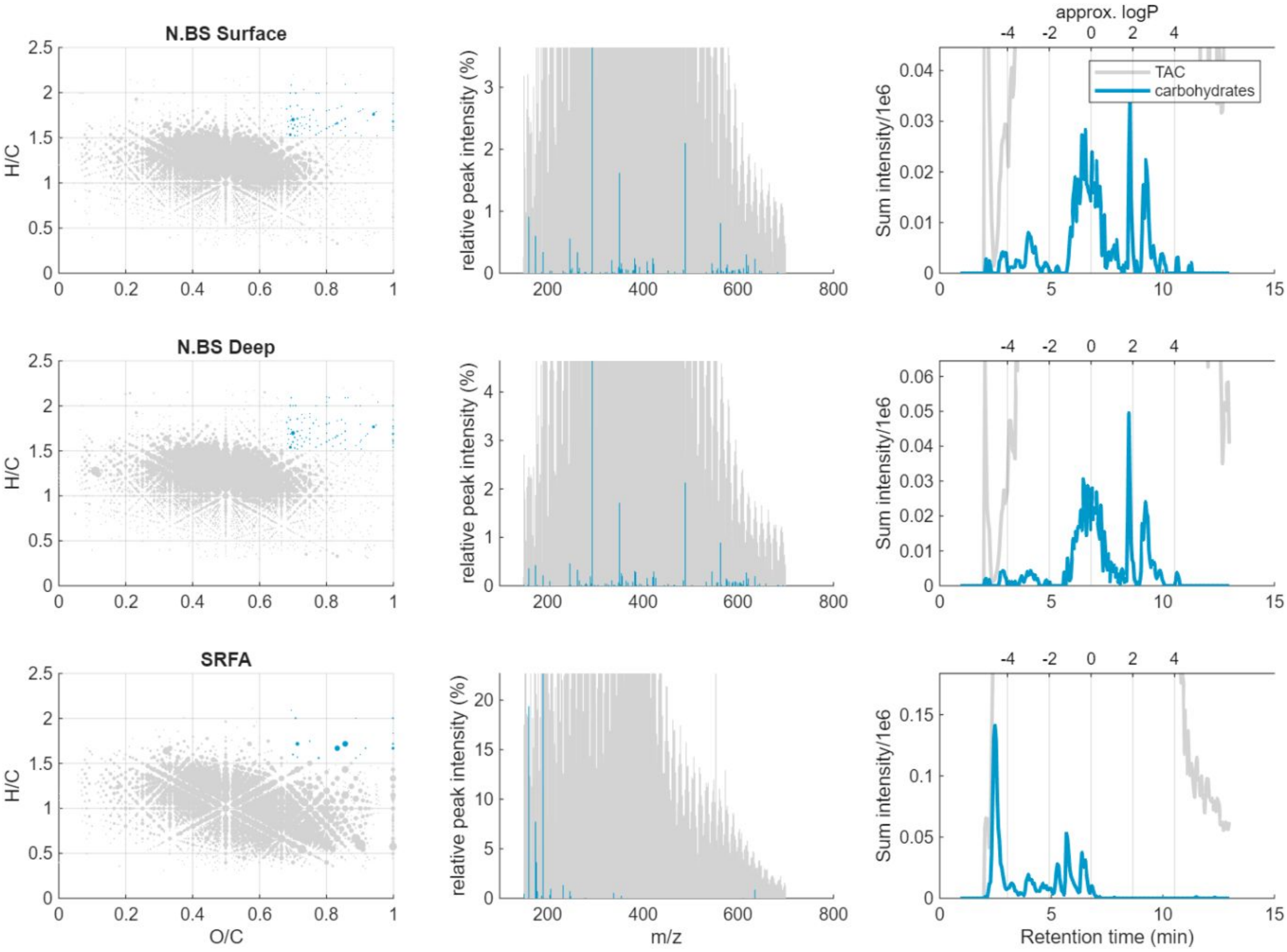

Figure SI5: Van Krevelen diagrams, mass spectra and retention profiles for the 'carbohydrate' compound class, superimposed onto all assigned formulas, assigned mass spectrum, and total ion current, respectively. The data are shown for all three samples, and in some cases the mass spectrum or TIC is out of scale, but can be seen in other figures (SI2-12).

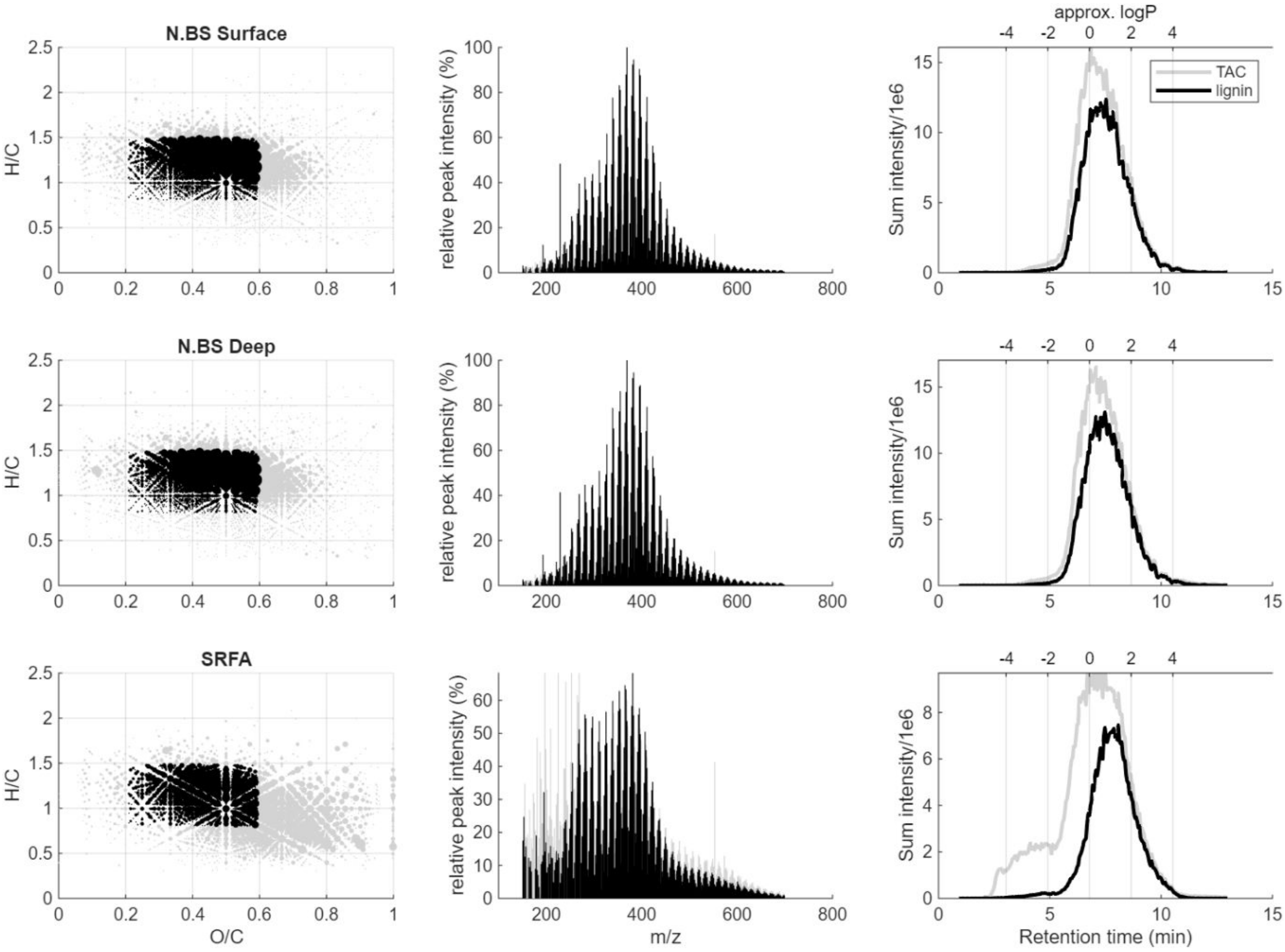

Figure SI6: Van Krevelen diagrams, mass spectra and retention profiles for the ‘lignin’ compound class, superimposed onto all assigned formulas, assigned mass spectrum, and total ion current, respectively. The data are shown for all three samples, and in some cases the mass spectrum or TIC is out of scale, but can be seen in other figures (SI2-12).

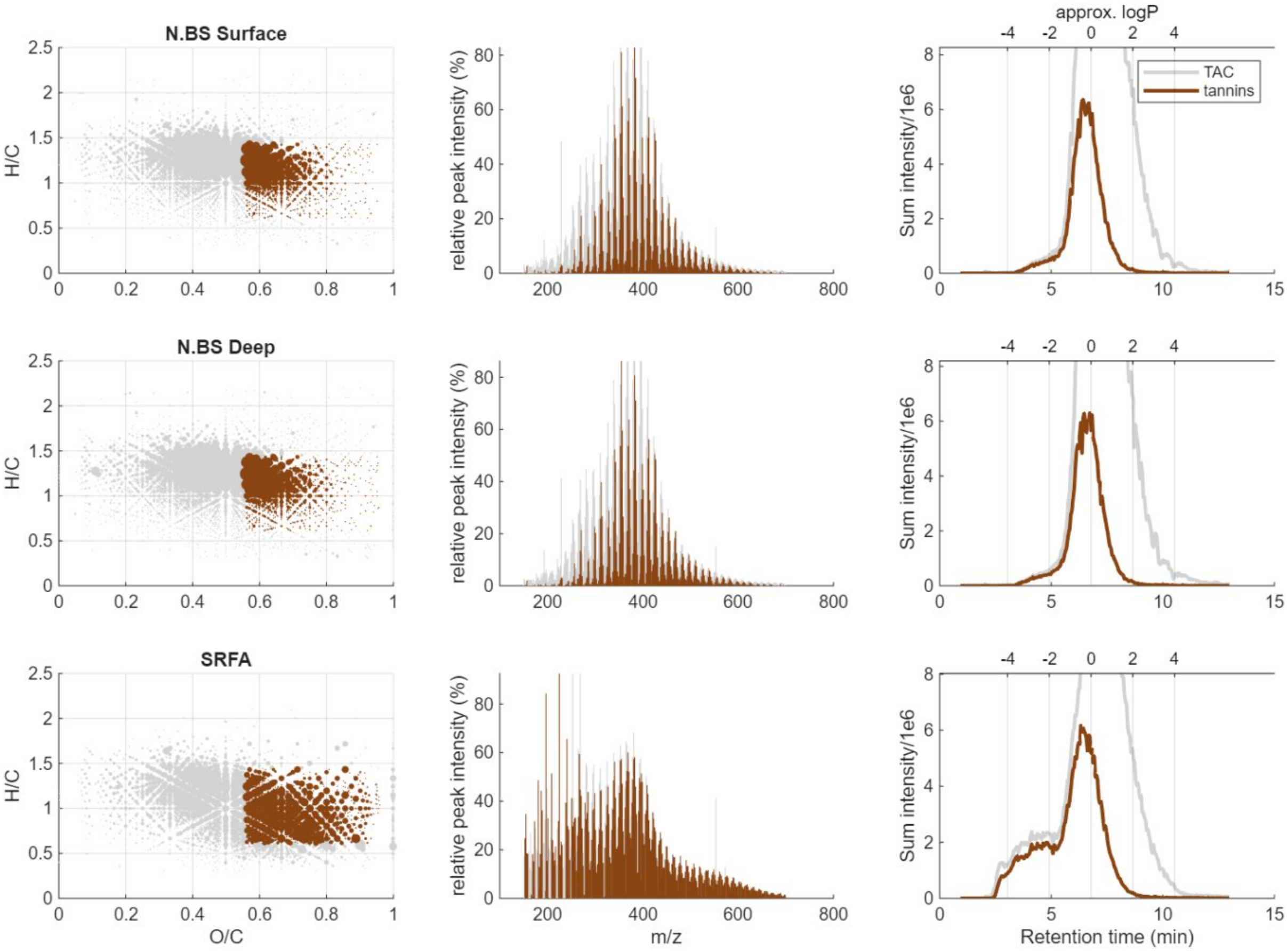

Figure SI7: Van Krevelen diagrams, mass spectra and retention profiles for the ‘tannin’ compound class, superimposed onto all assigned formulas, assigned mass spectrum, and total ion current, respectively. The data are shown for all three samples, and in some cases the mass spectrum or TIC is out of scale, but can be seen in other figures (SI2-12).

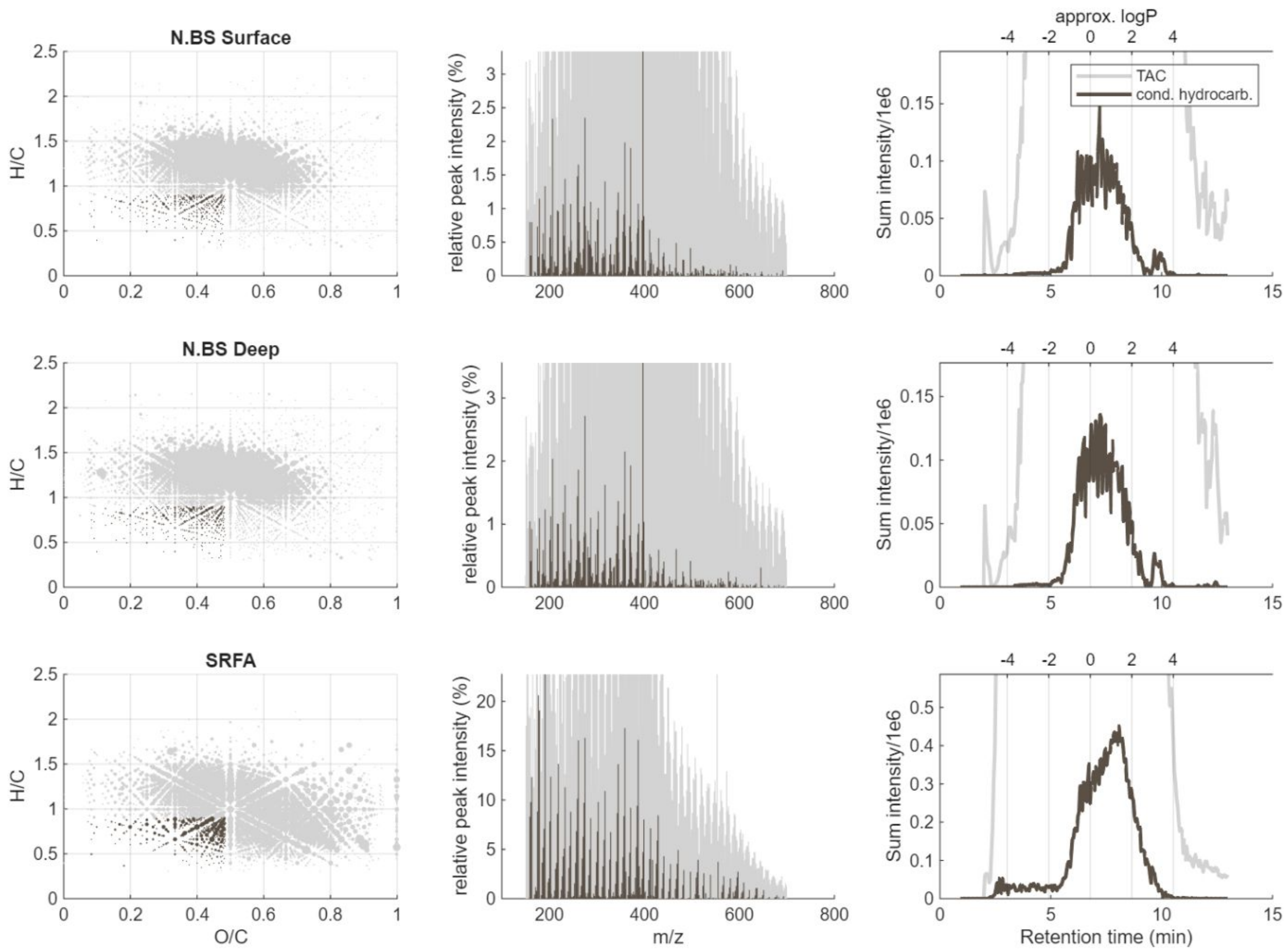

Figure SI8: Van Krevelen diagrams, mass spectra and retention profiles for the ‘condensed hydrocarbon’ compound class, superimposed onto all assigned formulas, assigned mass spectrum, and total ion current, respectively. The data are shown for all three samples, and in some cases the mass spectrum or TIC is out of scale, but can be seen in other figures (SI2-12).

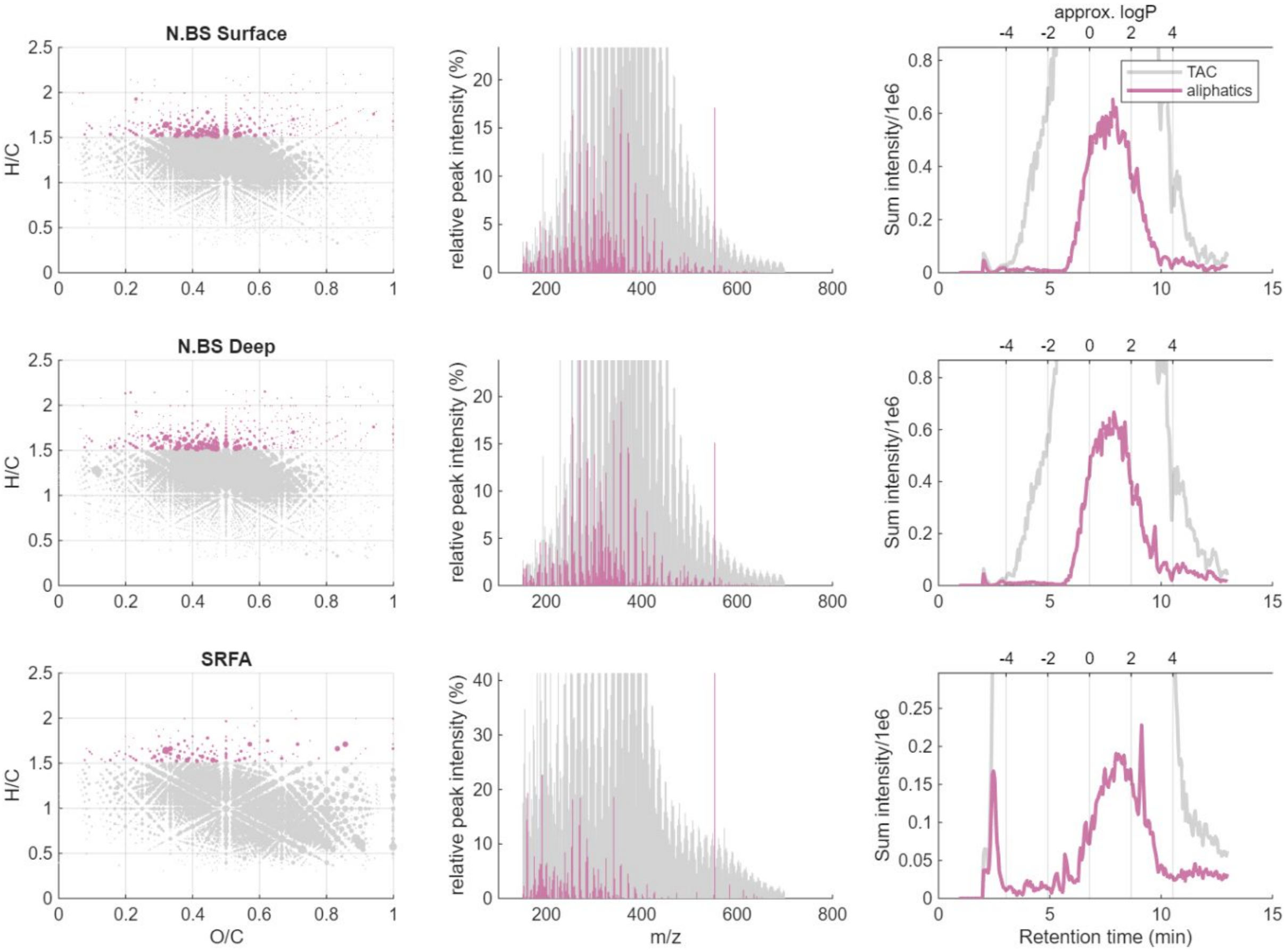

Figure SI9: Van Krevelen diagrams, mass spectra and retention profiles for the ‘aliphatics’ compound class, superimposed onto all assigned formulas, assigned mass spectrum, and total ion current, respectively. The data are shown for all three samples, and in some cases the mass spectrum or TIC is out of scale, but can be seen in other figures (SI2-12).

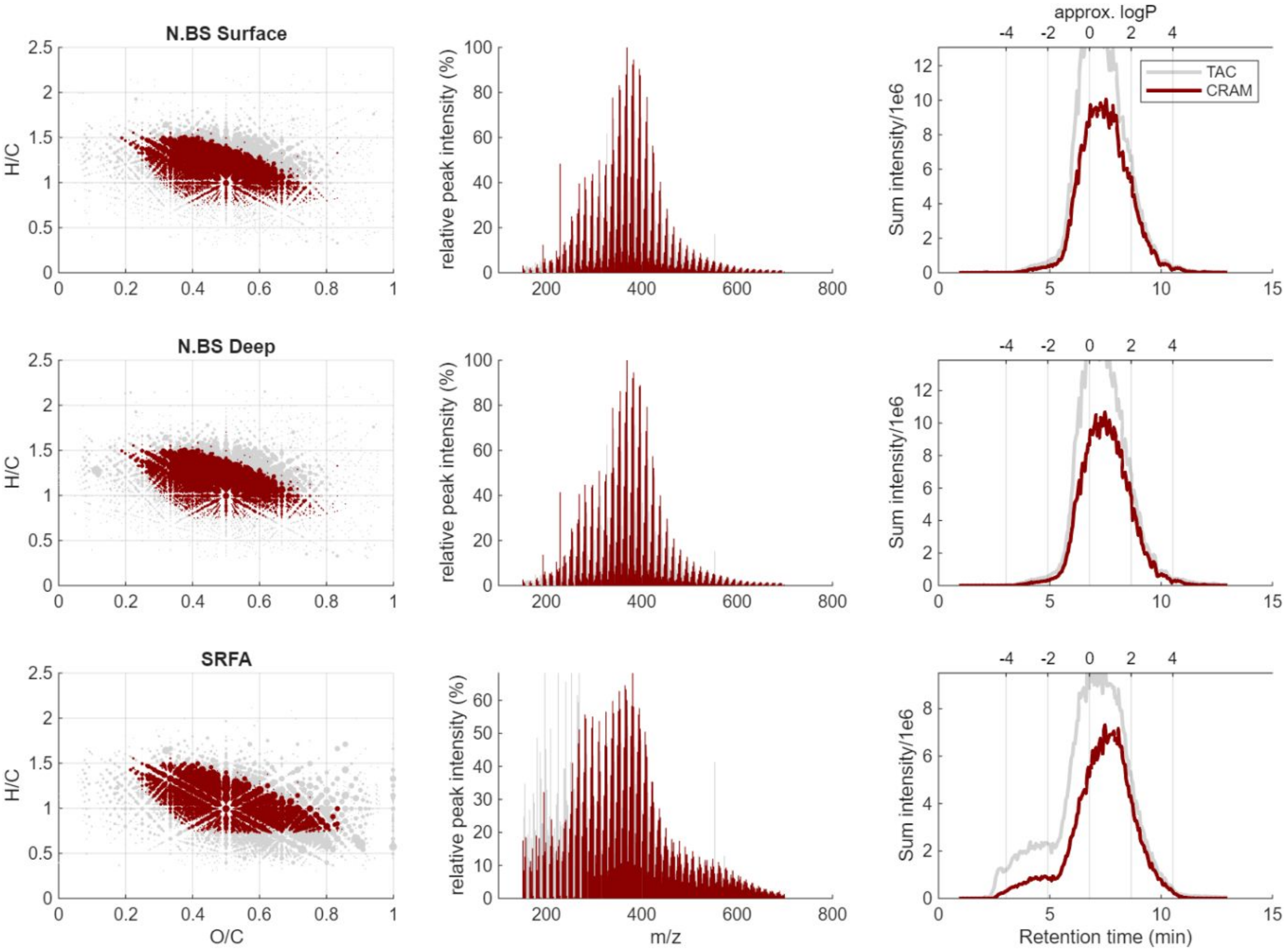

Figure SI10: Van Krevelen diagrams, mass spectra and retention profiles for the 'CRAM' compound class, superimposed onto all assigned formulas, assigned mass spectrum, and total ion current, respectively. The data are shown for all three samples, and in some cases the mass spectrum or TIC is out of scale, but can be seen in other figures (SI2-12).

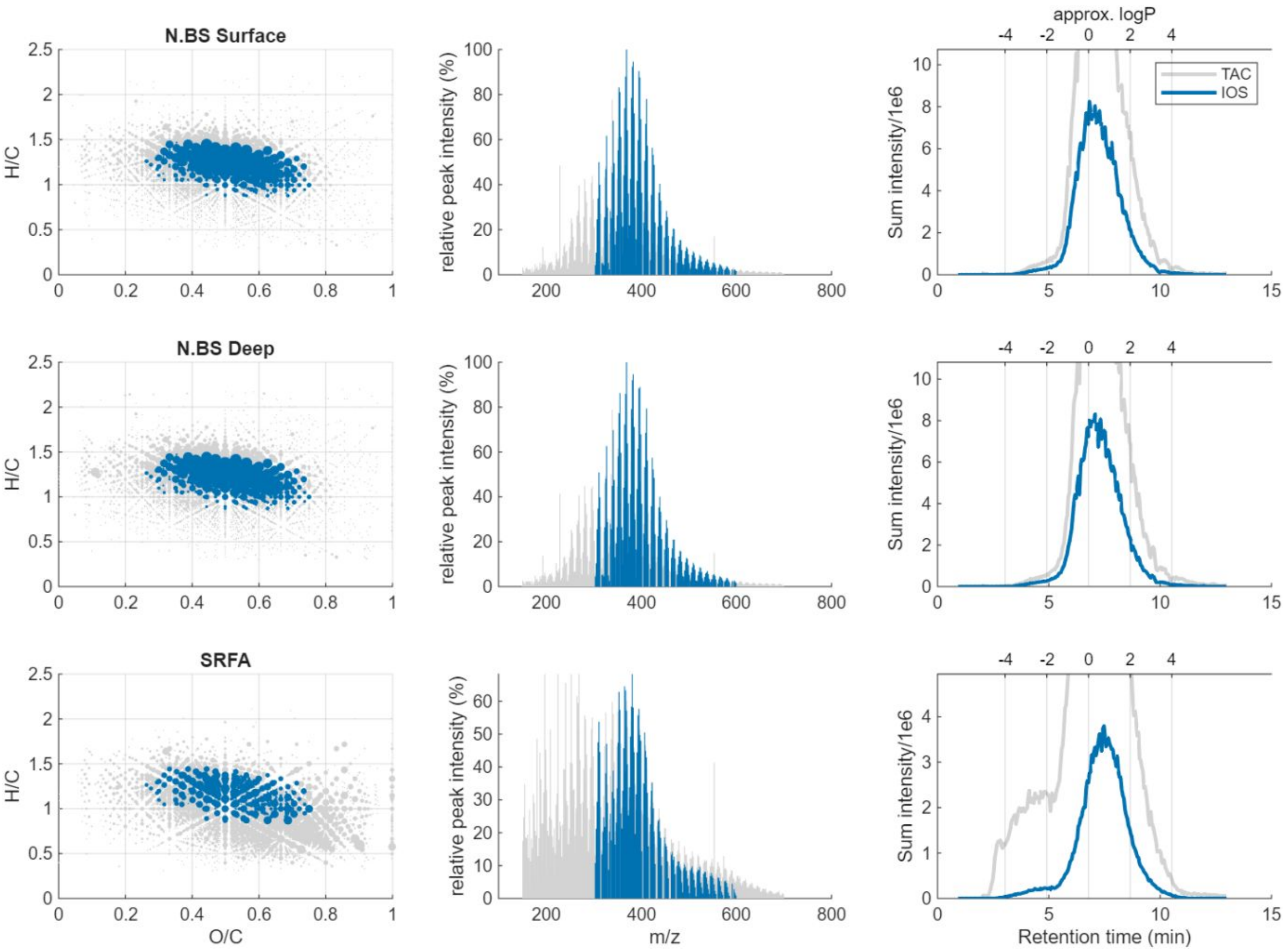

Figure SI11: Van Krevelen diagrams, mass spectra and retention profiles for the 'Island of Stability' formulas, superimposed onto all assigned formulas, assigned mass spectrum, and total ion current, respectively. The data are shown for all three samples, and in some cases the mass spectrum or TIC is out of scale, but can be seen in other figures (SI2-12).

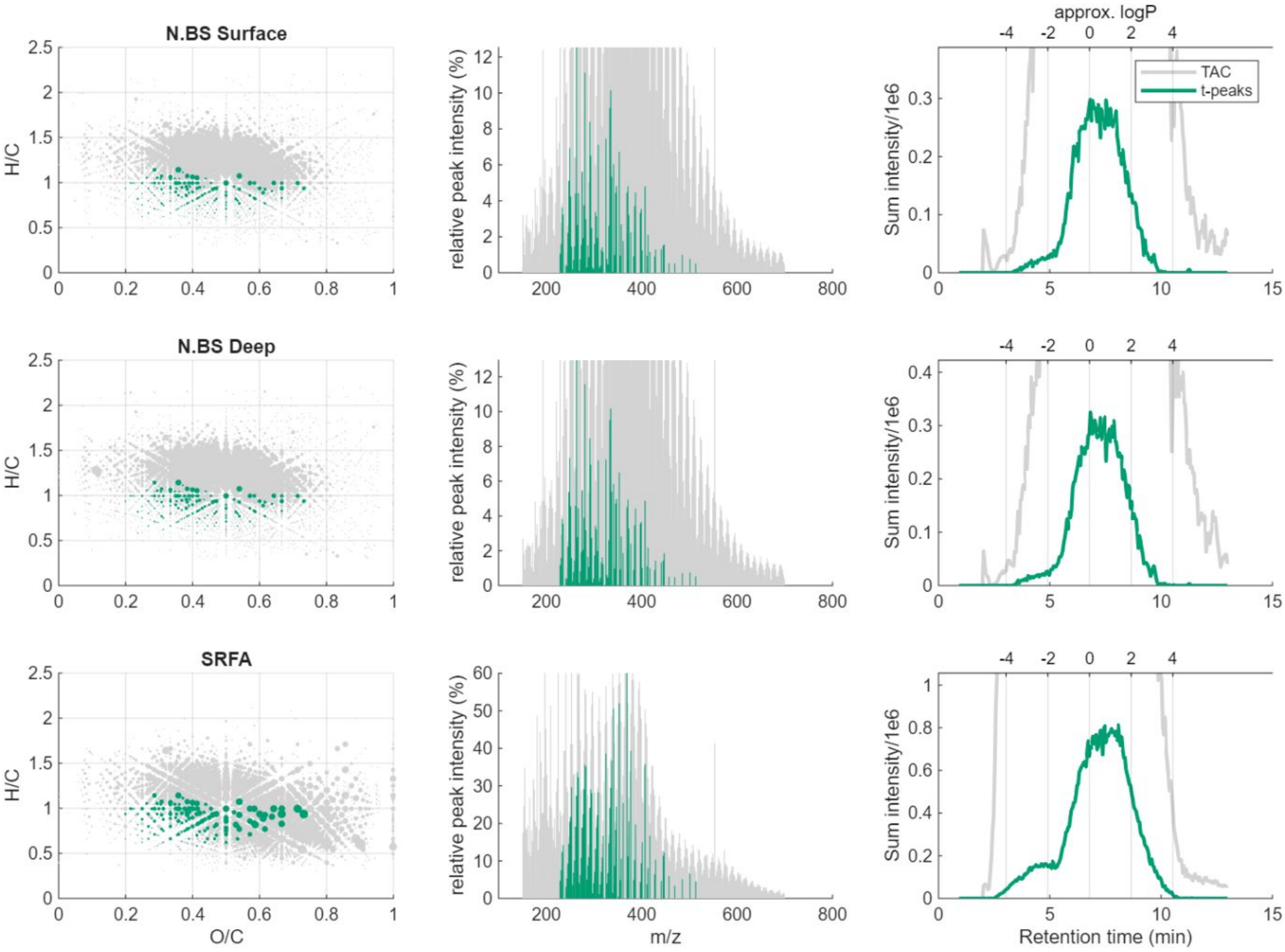

Figure SI12: Van Krevelen diagrams, mass spectra and retention profiles for the 't-peak' formulas, superimposed onto all assigned formulas, assigned mass spectrum, and total ion current, respectively. The data are shown for all three samples, and in some cases the mass spectrum or TIC is out of scale, but can be seen in other figures (SI2-12).

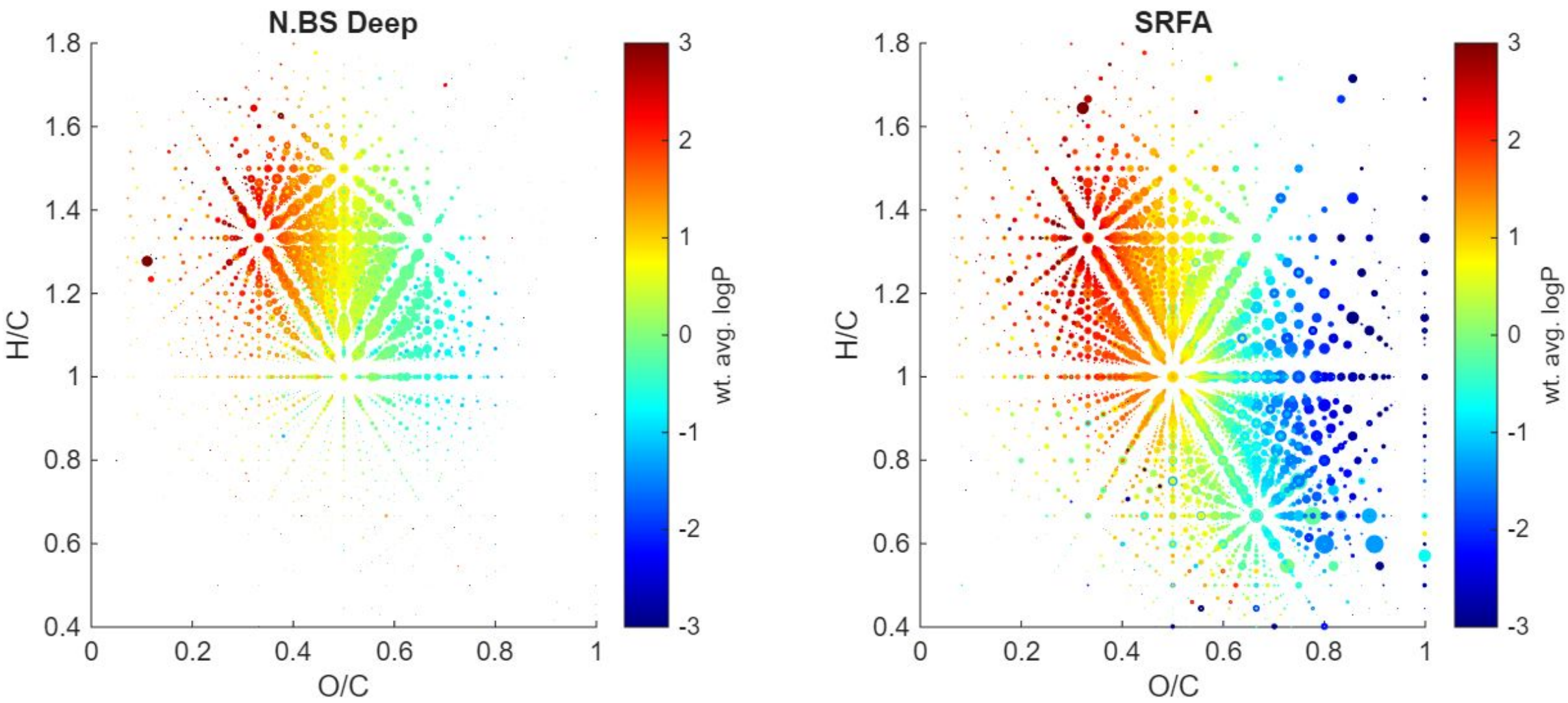

Figure SI13: Relationship between estimated logP and van Krevelen space. Data is shown for two samples (see titles), and each detected molecular formula is shown as a point in O/C vs H/C space, with point size as average intensity across the LC gradient, and point colour as weight averaged estimated logP.

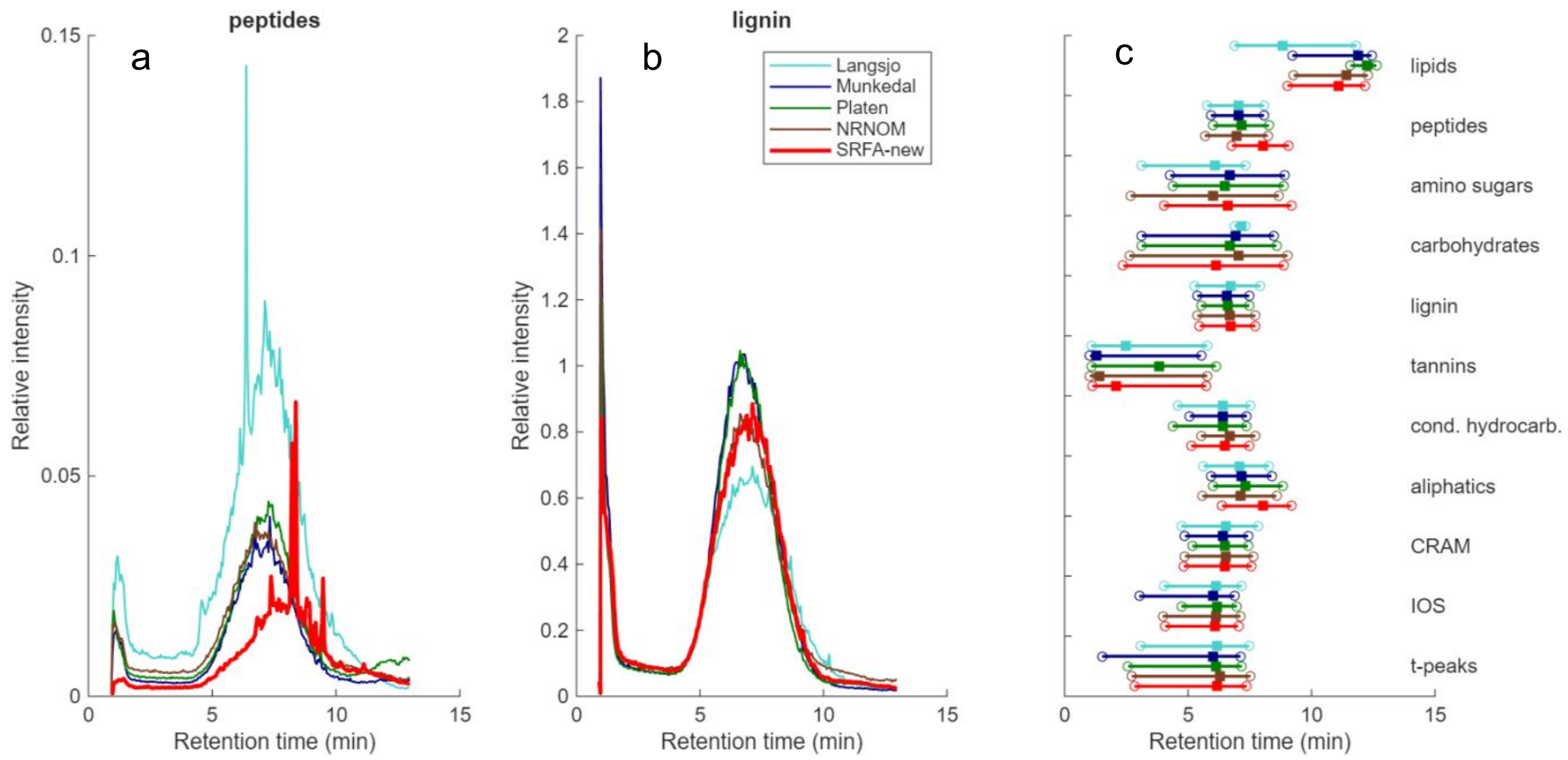

Figure SI14: Comparison of SRFA with four other terrestrial DOM samples. a) comparison of ‘peptides’, which are dissimilar between samples, with higher abundance and lower retention time in longer water residence time waters, b) comparison of ‘lignin’, which is similar in profile across the samples. c) comparison of 25th-75th percentile (unfilled circles) and median (squares) of each compound class across the five samples, showing general similarity.

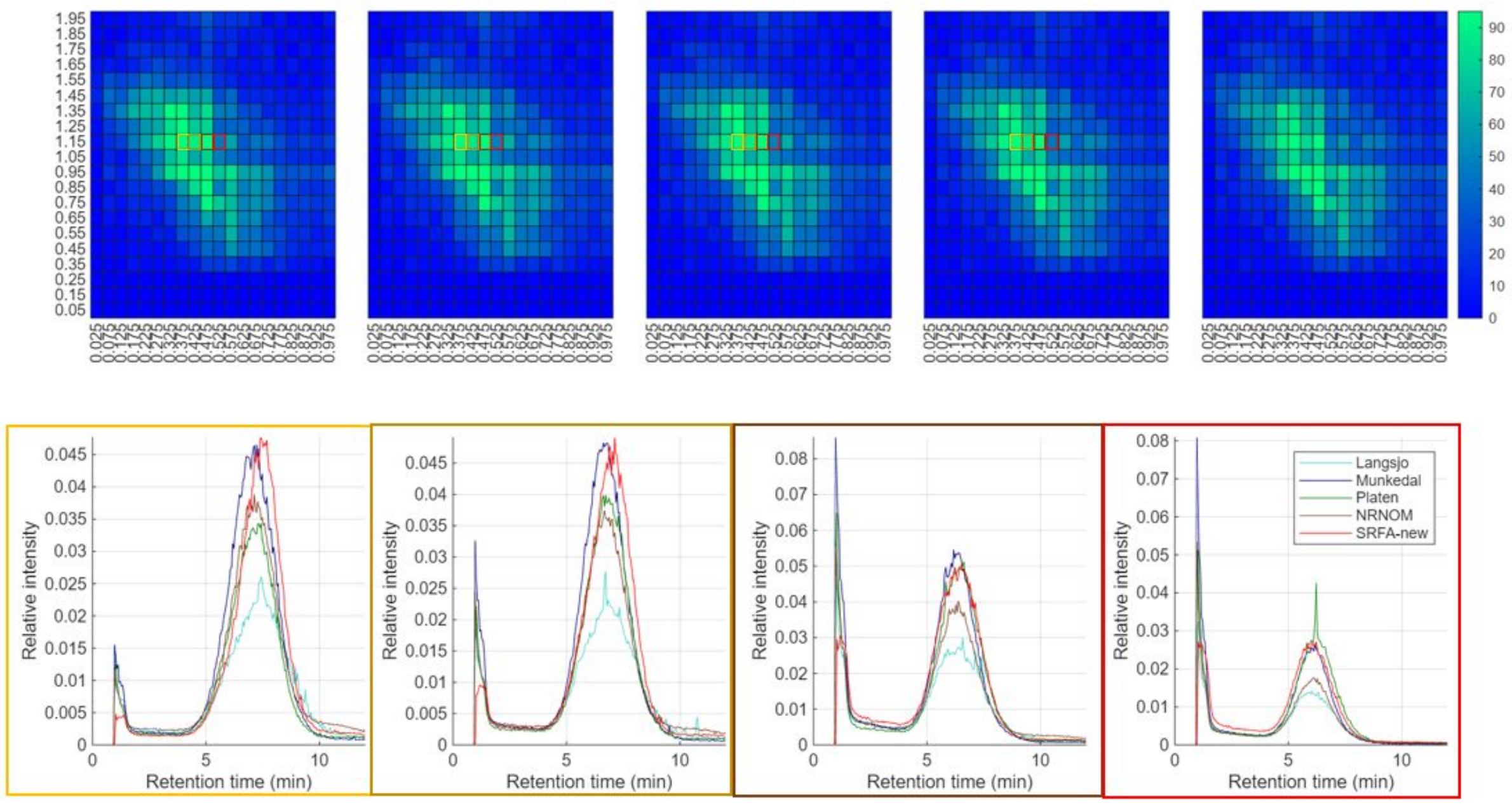

Figure SI15: Comparison of four adjacent grids in van Krevelen space for five terrestrial samples, showing general similarity in elution profile, but differing relative abundances in different sites.
